# Low-frequency variation in *TP53* has large effects on head circumference and intracranial volume

**DOI:** 10.1038/s41467-018-07863-x

**Published:** 2019-01-21

**Authors:** Simon Haworth, Chin Yang Shapland, Caroline Hayward, Bram P. Prins, Janine F. Felix, Carolina Medina-Gomez, Fernando Rivadeneira, Carol Wang, Tarunveer S. Ahluwalia, Martine Vrijheid, Mònica Guxens, Jordi Sunyer, Ioanna Tachmazidou, Klaudia Walter, Valentina Iotchkova, Andrew Jackson, Louise Cleal, Jennifer Huffmann, Josine L. Min, Lærke Sass, Paul R. H. J. Timmers, UK10K consortium, Saeed Al Turki, Carl A. Anderson, Richard Anney, Dinu Antony, María Soler Artigas, Muhammad Ayub, Senduran Bala, Jeffrey C. Barrett, Inês Barroso, Phil Beales, Jamie Bentham, Shoumo Bhattacharya, Ewan Birney, Douglas Blackwood, Martin Bobrow, Elena Bochukova, Patrick F. Bolton, Rebecca Bounds, Chris Boustred, Gerome Breen, Mattia Calissano, Keren Carss, Ruth Charlton, Krishna Chatterjee, Lu Chen, Antonio Ciampi, Sebahattin Cirak, Peter Clapham, Gail Clement, Guy Coates, Massimiliano Cocca, David A. Collier, Catherine Cosgrove, Tony Cox, Nick Craddock, Lucy Crooks, Sarah Curran, David Curtis, Allan Daly, Petr Danecek, Ian N. M. Day, Aaron Day-Williams, Anna Dominiczak, Thomas Down, Yuanping Du, Ian Dunham, Richard Durbin, Sarah Edkins, Rosemary Ekong, Peter Ellis, David M. Evans, I. Sadaf Farooqi, David R. Fitzpatrick, Paul Flicek, James Floyd, A. Reghan Foley, Christopher S. Franklin, Marta Futema, Louise Gallagher, Tom R. Gaunt, Matthias Geihs, Daniel Geschwind, Celia M. T. Greenwood, Heather Griffin, Detelina Grozeva, Xiaosen Guo, Xueqin Guo, Hugh Gurling, Deborah Hart, Audrey E. Hendricks, Peter Holmans, Bryan Howie, Jie Huang, Liren Huang, Tim Hubbard, Steve E. Humphries, Matthew E. Hurles, Pirro Hysi, David K. Jackson, Yalda Jamshidi, Chris Joyce, Konrad J. Karczewski, Jane Kaye, Thomas Keane, John P. Kemp, Karen Kennedy, Alastair Kent, Julia Keogh, Farrah Khawaja, Margriet van Kogelenberg, Anja Kolb-Kokocinski, Genevieve Lachance, Cordelia Langford, Daniel Lawson, Irene Lee, Monkol Lek, Rui Li, Yingrui Li, Jieqin Liang, Hong Lin, Ryan Liu, Jouko Lönnqvist, Luis R. Lopes, Margarida Lopes, Daniel G. MacArthur, Massimo Mangino, Jonathan Marchini, Gaëlle Marenne, John Maslen, Iain Mathieson, Shane McCarthy, Peter McGuffin, Andrew M. McIntosh, Andrew G. McKechanie, Andrew McQuillin, Yasin Memari, Sarah Metrustry, Nicola Migone, Hannah M. Mitchison, Alireza Moayyeri, Andrew Morris, James Morris, Dawn Muddyman, Francesco Muntoni, Kate Northstone, Michael C. O’Donovan, Stephen O’Rahilly, Alexandros Onoufriadis, Karim Oualkacha, Michael J. Owen, Aarno Palotie, Kalliope Panoutsopoulou, Victoria Parker, Jeremy R. Parr, Lavinia Paternoster, Tiina Paunio, Felicity Payne, Stewart J. Payne, John R. B. Perry, Olli Pietilainen, Vincent Plagnol, Rebecca C. Pollitt, David J. Porteous, Sue Povey, Michael A. Quail, Lydia Quaye, F. Lucy Raymond, Karola Rehnström, J. Brent Richards, Cheryl K. Ridout, Susan Ring, Graham R. S. Ritchie, Nicola Roberts, Rachel L. Robinson, David B. Savage, Peter Scambler, Stephan Schiffels, Miriam Schmidts, Nadia Schoenmakers, Richard H. Scott, Robert K. Semple, Eva Serra, Sally I. Sharp, Adam Shaw, Hashem A. Shihab, So-Youn Shin, David Skuse, Kerrin S. Small, Carol Smee, Blair H. Smith, Nicole Soranzo, Lorraine Southam, Olivera Spasic-Boskovic, Timothy D. Spector, David St Clair, Jim Stalker, Elizabeth Stevens, Jianping Sun, Gabriela Surdulescu, Jaana Suvisaari, Petros Syrris, Rohan Taylor, Jing Tian, Martin D. Tobin, Ana M. Valdes, Anthony M. Vandersteen, Parthiban Vijayarangakannan, Peter M. Visscher, Louise V. Wain, James T. R. Walters, Guangbiao Wang, Jun Wang, Yu Wang, Kirsten Ward, Eleanor Wheeler, Tamieka Whyte, Hywel J. Williams, Kathleen A. Williamson, Crispian Wilson, Scott G. Wilson, Kim Wong, ChangJiang Xu, Jian Yang, Feng Zhang, Pingbo Zhang, Hou-Feng Zheng, George Davey Smith, Simon E. Fisher, James F. Wilson, Tim J. Cole, Dietmar Fernandez-Orth, Klaus Bønnelykke, Hans Bisgaard, Craig E. Pennell, Vincent W. V. Jaddoe, George Dedoussis, Nicholas Timpson, Eleftheria Zeggini, Veronique Vitart, Beate St Pourcain

**Affiliations:** 1 MRC Integrative Epidemiology Unit, Department of Population Health Sciences, Bristol Medical School, University of Bristol, Bristol, BS8 2BN UK; 2 Language and Genetics Department, Max Planck Institute for Psycholinguistics, 6525 XD Nijmegen, The Netherlands; 3 MRC Human Genetics Unit, MRC Institute of Genetics and Molecular Medicine, University of Edinburgh, Edinburgh, EH4 2XU UK; 4 Wellcome Sanger Institute, Wellcome Genome Campus, Hinxton, CB10 1SA UK; 5 The Generation R Study Group, Erasmus MC, University Medical Center Rotterdam, 3000 CA Rotterdam, The Netherlands; 6 Department of Epidemiology, Erasmus MC, University Medical Center Rotterdam, 3000 CA Rotterdam, The Netherlands; 7 Department of Pediatrics, Erasmus MC, University Medical Center Rotterdam, 3000 CA Rotterdam, The Netherlands; 8 Department of Internal Medicine, Erasmus MC, University Medical Center Rotterdam, 3000 CA Rotterdam, The Netherlands; 9 School of Medicine and Public Health, Faculty of Medicine and Health, The University of Newcastle, Newcastle, NSW 2308 Australia; 10 Division of Obstetrics and Gynaecology, The University of Western Australia, Crawley, WA 6009 Australia; 11 COPSAC, Copenhagen Prospective Studies on Asthma in Childhood, Herlev and Gentofte Hospital, University of Copenhagen, 2820 Copenhagen, Denmark; 12 ISGlobal, 08003 Barcelona, Spain; 13 Pompeu Fabra University, Barcelona, 08003 Spain; 14 Spanish Consortium for Research on Epidemiology and Public Health, Instituto de Salud Carlos III, Madrid, 28029 Spain; 15 Department of Child and Adolescent Psychiatry/Psychology, Erasmus University Medical Centre-Sophia Children’s Hospital, P.O. Box 2060, Rotterdam, 3000 CB The Netherlands; 16 IMIM Instituto Hospital del Mar de Investigaciones Médicas, Barcelona, 08003 Spain; 17 MRC Weatherall Institute of Molecular Medicine, University of Oxford, Oxford, OX3 9DS UK; 18 Center for Population Genomics, Boston VA Healthcare System, 150 S. Huntington Ave, Jamaica Plain, MA 02130 USA; 19 Centre for Global Health Research, Usher Institute for Population Health Sciences and Informatics, University of Edinburgh, Edinburgh, EH8 9AG UK; 20 Donders Institute for Brain, Cognition & Behaviour, Radboud University, 6525 EN Nijmegen, The Netherlands; 21 Faculty of Population Health Sciences, UCL Great Ormond Street Institute of Child Health, London, WC1N 1EH UK; 22 Department of Nutrition and Dietetics, School of Health Science and Education, Harokopio University, 17671 Athens, Greece; 23 Department of Pathology, King Abdulaziz Medical City, P.O. Box 22490, Riyadh, 11426 Saudi Arabia; 24 Department of Psychiatry, Trinity Centre for Health Sciences, St James Hospital, James Street, Dublin, 8 Ireland; 25 Genetics and Genomic Medicine and Birth Defects Research Centre, UCL Institute of Child Health, London, WC1N 1EH UK; 26 Departments of Health Sciences and Genetics, University of Leicester, Leicester, LE1 7RH UK; 27 Division of Developmental Disabilities, Department of Psychiatry, Queen’s University, Kingston, ON N6C 0A7 Canada; 28 University of Cambridge Metabolic Research Laboratories, and NIHR Cambridge Biomedical Research Centre, Wellcome Trust-MRC Institute of Metabolic Science, Addenbrooke’s Hospital, Cambridge, CB2 0QQ UK; 29 Department of Cardiovascular Medicine and Wellcome Trust Centre for Human Genetics, Roosevelt Drive, Oxford, OX3 7BN UK; 30 European Molecular Biology Laboratory, European Bioinformatics Institute, Wellcome Trust Genome Campus, Hinxton, Cambridge, CB10 1SD UK; 31 Division of Psychiatry, The University of Edinburgh, Royal Edinburgh Hospital, Edinburgh, EH10 5HF UK; 32 Academic Laboratory of Medical Genetics, Box 238, Lv 6 Addenbrooke’s Treatment Centre, Addenbrooke’s Hospital, Cambridge, CB2 0QQ UK; 33 Department of Child Psychiatry, Institute of Psychiatry, Psychology and Neuroscience, King’s College London, 16 De Crespigny Park, London, SE5 8AF UK; 34 NIHR BRC for Mental Health, Institute of Psychiatry, Psychology and Neuroscience and SLaM NHS Trust, King’s College London, 16 De Crespigny Park, London, SE5 8AF UK; 35 MRC Social, Genetic and Developmental Psychiatry Centre, Institute of Psychiatry, Psychology and Neuroscience, King’s College London, Denmark Hill, London, SE5 8AF UK; 36 North East Thames Regional Genetics Service, Great Ormond Street Hospital NHS Foundation Trust, London, WC1N 3JH UK; 37 Dubowitz Neuromuscular Centre, UCL Institute of Child Health & Great Ormond Street Hospital, London, WC1N 1EH UK; 38 Leeds Genetics Laboratory, St James University Hospital, Beckett Street, Leeds, LS9 7TF UK; 39 Department of Haematology, University of Cambridge, Long Road, Cambridge, CB2 0PT UK; 40 Department of Epidemiology, Biostatistics and Occupational Health, McGill University, Montreal, QC H3A 1A2 Canada; 41 Institut für Humangenetik, Uniklinik Köln, Kerpener Strasse 34, 50931 Köln, Germany; 42 The Department of Twin Research & Genetic Epidemiology, King’s College London, St Thomas’ Campus, Lambeth Palace Road, London, SE1 7EH UK; 43 Medical Genetics, Institute for Maternal and Child Health IRCCS “Burlo Garofolo”, 34100 Trieste, Italy; 44 Department of Medical, Surgical and Health Sciences, University of Trieste, 34100 Trieste, Italy; 45 Lilly Research Laboratories, Eli Lilly & Co. Ltd., Erl Wood Manor, Sunninghill Road, Windlesham, GU20 6PH UK; 46 MRC Centre for Neuropsychiatric Genetics & Genomics, Institute of Psychological Medicine & Clinical Neurosciences, School of Medicine, Cardiff University, Cardiff, CF24 4HQ UK; 47 Sheffield Diagnostic Genetics Service, Sheffield Childrens’ NHS Foundation Trust, Western Bank, Sheffield, S10 2TH UK; 48 University of Sussex, Brighton, BN1 9RH UK; 49 Sussex Partnership NHS Foundation Trust, Swandean, Arundel Road, Worthing, BN13 3EP UK; 50 UCL Genetics Institute, University College London (UCL), Darwin Building, Gower Street, London, WC1E 6BT UK; 51 Bristol Genetic Epidemiology Laboratories, School of Social and Community Medicine, University of Bristol, Oakfield House, Oakfield Grove, Clifton, Bristol, BS8 2BN UK; 52 Computational Biology & Genomics, Biogen Idec, 14 Cambridge Center, Cambridge, MA 02142 USA; 53 Institute of Cardiovascular and Medical Sciences, University of Glasgow, Wolfson Medical School Building, University Avenue, Glasgow, G12 8QQ UK; 54 Department of Medical and Molecular Genetics, Division of Genetics and Molecular Medicine, King’s College London School of Medicine, Guy’s Hospital, London, SE1 9RT UK; 55 BGI-Shenzhen, 518083 Shenzhen, China; 56 University College London (UCL) Department of Genetics, Evolution & Environment (GEE), Gower Street, London, WC1E 6BT UK; 57 University of Queensland Diamantina Institute, Translational Research Institute, Brisbane, QLD 4102 Australia; 58 The Genome Centre, John Vane Science Centre, Queen Mary, University of London, Charterhouse Square, London, EC1M 6BQ UK; 59 Cardiovascular Genetics, BHF Laboratories, Rayne Building, Institute of Cardiovascular Sciences, University College London, London, WC1E 6JJ UK; 60 UCLA David Geffen School of Medicine, Los Angeles, CA 90095 USA; 61 Lady Davis Institute, Jewish General Hospital, Montreal, QC H3T 1E2 Canada; 62 Department of Human Genetics, McGill University, Montreal, QC H3A 1B1 Canada; 63 Department of Oncology, McGill University, Montreal, QC H2W 1S6 Canada; 64 HeLEX—Centre for Health, Law and Emerging Technologies, Nuffield Department of Population Health, University of Oxford, Old Road Campus, Oxford, OX3 7LF UK; 65 Department of Biology, University of Copenhagen, Ole Maaløes Vej 5, 2200 Copenhagen, Denmark; 66 Molecular Psychiatry Laboratory, Division of Psychiatry, University College London (UCL), Gower Street, London, WC1E 6BT UK; 67 Department of Mathematical and Statistical Sciences, University of Colorado, Denver, CO 80204 USA; 68 Adaptive Biotechnologies Corporation, Seattle, WA 98102 USA; 69 Human Genetics Research Centre, St George’s University of London, London, SW17 0RE UK; 70 Analytic and Translational Genetics Unit, Massachusetts General Hospital, Boston, MA 02114 USA; 71 Program in Medical and Population Genetics, Broad Institute of Harvard and MIT, Cambridge, MA 02142 USA; 72 National Cancer Research Institute, Angel Building, 407 St John Street, London, EC1V 4AD UK; 73 Genetic Alliance UK, 4D Leroy House, 436 Essex Road, London, N1 3QP UK; 74 SW Thames Regional Genetics Lab, St George’s University, Cranmer Terrace, London, SW17 0RE UK; 75 Schools of Mathematics and Social and Community Medicine, University of Bristol, Oakfield House, Oakfield Grove, Clifton, Bristol, BS8 2BN UK; 76 Behavioural and Brain Sciences Unit, UCL Institute of Child Health, London, WC1N 1EH UK; 77 Department of Medicine, Jewish General Hospital, McGill University, Montreal, QC H3A 1B1 Canada; 78 BGI-Europe, London, EC2M 4YE UK; 79 National Institute for Health and Welfare (THL), FI-00271 Helsinki, Finland; 80 Institute of Cardiovascular Science, University College London, Gower Street, London, WC1E 6BT UK; 81 Cardiovascular Centre of the University of Lisbon, Faculty of Medicine, University of Lisbon, Avenida Professor Egas Moniz, 1649-028 Lisbon, Portugal; 82 Wellcome Trust Centre for Human Genetics, Roosevelt Drive, Oxford, OX3 7BN UK; 83 Illumina Cambridge Ltd, Chesterford Research Park, Cambridge, CB10 1XL UK; 84 National Institute for Health Research (NIHR) Biomedical Research Centre at Guy’s and St Thomas’ Foundation Trust, London, SE1 9RT UK; 85 Department of Statistics, University of Oxford, 1 South Parks Road, Oxford, OX1 3TG UK; 86 Department of Genetics, Harvard Medical School, Boston, MA 02115 USA; 87 The Patrick Wild Centre, The University of Edinburgh, Edinburgh, EH10 5HF UK; 88 Department of Medical Sciences, University of Torino, 10124 Torino, Italy; 89 Institute of Health Informatics, Farr Institute of Health Informatics Research, University College London (UCL), 222 Euston Road, London, NW1 2DA UK; 90 Department of Mathematics, Université de Québec À Montréal, Montréal, QC H3C 3P8 Canada; 91 Institute for Molecular Medicine Finland (FIMM), University of Helsinki, FI-00014 Helsinki, Finland; 92 Program in Medical and Population Genetics and Genetic Analysis Platform, The Broad Institute of MIT and Harvard, Cambridge, MA 02132 USA; 93 Institute of Neuroscience, Henry Wellcome Building for Neuroecology, Newcastle University, Framlington Place, Newcastle upon Tyne, NE2 4HH UK; 94 Department of Psychiatry, University of Helsinki, FI-00014 Helsinki, Finland; 95 North West Thames Regional Genetics Service, Kennedy-Galton Centre, Northwick Park Hospital, Watford Road, Harrow, HA1 3UJ UK; 96 MRC Epidemiology Unit, University of Cambridge School of Clinical Medicine, Box 285, Institute of Metabolic Science, Cambridge Biomedical Campus, Cambridge, CB2 0QQ UK; 97 University College London (UCL) Genetics Institute (UGI), Gower Street, London, WC1E 6BT UK; 98 Connective Tissue Disorders Service, Sheffield Diagnostic Genetics Service, Sheffield Children’s NHS Foundation Trust, Western Bank, Sheffield, S10 2TH UK; 99 Centre for Genomic and Experimental Medicine, Institute of Genetics and Experimental Medicine, University of Edinburgh, Western General Hospital, Crewe Road, Edinburgh, EH4 2XU UK; 100 Molecular Genetics, Viapath at Guy’s Hospital, London, SE1 9RT UK; 101 ALSPAC & School of Social and Community Medicine, University of Bristol, Oakfield House, Oakfield Grove, Clifton, Bristol, BS8 2BN UK; 102 Human Genetics Department, Radboudumc and Radboud Institute for Molecular Life Sciences (RIMLS), Geert Grooteplein 25, 6525 HP Nijmegen, The Netherlands; 103 Department of Clinical Genetics, Great Ormond Street Hospital, London, WC1N 3JH UK; 104 Clinical Genetics, Guy’s & St Thomas’ NHS Foundation Trust, London, SE1 9RT UK; 105 Ninewells Hospital and Medical School, Mackenzie Building, Kirsty Semple Way, Dundee, DD2 4RB UK; 106 Institute of Medical Sciences, University of Aberdeen, Aberdeen, AB25 2ZD UK; 107 National Institute for Health Research (NIHR) Leicester Respiratory Biomedical Research Unit, Glenfield Hospital, Leicester, LE3 9QP UK; 108 Maritime Medical Genetics Service, 5850/5980 University Avenue, PO Box 9700, Halifax, NS B3K 6R8 Canada; 109 Queensland Brain Institute, University of Queensland, Brisbane, QLD 4072 Australia; 110 Princess Al Jawhara Albrahim Center of Excellence in the Research of Hereditary Disorders, King Abdulaziz University, P.O. Box 80200, Jeddah, 21589 Saudi Arabia; 111 Macau University of Science and Technology, Avenida Wai long, Taipa, Macau, 999078 China; 112 Department of Medicine and State Key Laboratory of Pharmaceutical Biotechnology, University of Hong Kong, 21 Sassoon Road, Hong Kong, Pokfulam Hong Kong; 113 The Centre for Translational Omics—GOSgene, UCL Institute of Child Health, London, WC1N 1EH UK; 114 School of Medicine and Pharmacology, University of Western Australia, Perth, WA 6009 Australia; 115 Department of Endocrinology and Diabetes, Sir Charles Gairdner Hospital, Nedlands, WA 6009 Australia

## Abstract

Cranial growth and development is a complex process which affects the closely related traits of head circumference (HC) and intracranial volume (ICV). The underlying genetic influences shaping these traits during the transition from childhood to adulthood are little understood, but might include both age-specific genetic factors and low-frequency genetic variation. Here, we model the developmental genetic architecture of HC, showing this is genetically stable and correlated with genetic determinants of ICV. Investigating up to 46,000 children and adults of European descent, we identify association with final HC and/or final ICV + HC at 9 novel common and low-frequency loci, illustrating that genetic variation from a wide allele frequency spectrum contributes to cranial growth. The largest effects are reported for low-frequency variants within *TP53*, with 0.5 cm wider heads in increaser-allele carriers versus non-carriers during mid-childhood, suggesting a previously unrecognized role of *TP53* transcripts in human cranial development.

## Abstract (web summary)

Size and shape of the brain are, among others, influenced by the dimensions of the skull. Here, the authors report genome-wide association studies for head circumference and intracranial volume in children and adults and the identification of nine common or low-frequency variants associated with these traits.

## Introduction

The size and shape of the vertebrate brain is governed by the internal dimensions of the skull. Across vertebrate evolutionary history, major changes to brain size and proportion have been accompanied by modifications to skull morphology^1,2^. This is also true within the lifespan of an individual, where developmental changes in brain size and shape must be reflected in changing cranial phenotypes.

Serial measures of maximal head circumference (HC) or occipito-frontal circumference are routinely obtained to monitor children’s cranial growth and brain development during the first years of life and abnormal trajectories may indicate a range of neurological conditions^3^. In infants and children, HC is highly correlated with brain volume as measured by MRI studies^4,5^, especially in 1.7- to 6-year-old children, although its predictive accuracy decreases with progressing age^5^. Healthy children from around the world, who are raised in healthy environments and follow recommended feeding practices, have strikingly similar patterns of growth^6^. The observation that final HC is largely determined by the age of 6 years in a large study from the UK^7^ is therefore likely to be valid in multiple populations. In addition, nutritional status, body size, and HC are closely correlated for healthy children during early life, and become less related after 24 months of age^8^. While HC properties in early childhood have immediate medical relevance, there are also compelling reasons to study HC in adulthood. In the adult population skeletal measures continue to act as a permanent measure of peak brain size that is unaffected by subsequent atrophic brain changes^9^. In early childhood, HC is likely to proxy overall body size and timing of growth, tracking changes in brain size. In older individuals, HC is valuable precisely because HC is robust to soft tissue atrophy, solely reflecting an absolute measure of final HC dimension.

HC is highly heritable and the notion of a developmentally changing, but etiologically interrelated, phenotypic expression of HC during the life course is supported by twin studies^10^. Reported twin-h^2^ estimates are 90% in infants, 85–88% in early childhood, 83–87% in adolescence and 75% in young and mid adulthood^10^, with evidence for strong genetic stability between mid-childhood and early adulthood^10^. There are arguments to support the hypothesis that some of the underlying genetic factors act by a coordinated integration of signaling pathways regulating both brain and skull morphogenesis during development^11^. Especially, cells of early brain and skull are sensitive to similar signaling families^11^. Genetic underpinning of potentially shared mechanisms is supported by the fact that genome-wide signals for both infant HC and intracranial volume (ICV) are strengthened when combined^12^, irrespective of their dissimilar developmental stages. However, genetic investigations studying (near) final HC and adult ICV are likely to be more informative on mechanisms underlying developmentally shared growth patterning which affect final cranial dimension. Additionally, low-frequency genetic variants, ranging between 0.5 to 5% minor allele frequency, have been poorly characterized by previous genome-wide association study (GWAS) efforts^13^, both due to the small size of previous studies, and the limited coverage of lower-frequency markers by the first imputation panels.

Exploiting whole-genome sequence data together with high-density imputation panels such as the joint UK10K and 1000 genomes (UK10K/1KGP)^14^ and the haplotype reference consortium (HRC)^15^, that have previously facilitated the discovery of low-frequency genetic variants for a range of traits^16,17^, we carry out GWAS for final HC. Specifically, we aim to

- study low-frequency and common variants for final HC, allowing for age-specific effects through meta-analyses of mid-childhood and/or adulthood datasets,
- investigate genetic variants influencing a combined phenotype of (near) final HC and ICV, termed final cranial dimension, and
- explore developmental changes in the genetic architecture of HC through longitudinal modeling of genetic variances in unrelated individuals as well as growth curve modeling of HC trajectories for carriers and noncarriers of high risk variants.

Through these analyses we show that the developmental genetic architecture of HC is genetically stable during the course of childhood and adolescence and correlates with genetic determinants of ICV. Integrating information from both (near) final HC and ICV in a combined analysis including up to 46,000 children and adults of European descent, we identify nine novel common and low-frequency loci for either HC or HC + ICV, including low-frequency variation within *TP53*. Collectively, these findings provide insight into the genetic effects influencing cranial growth during childhood and adolescence, while yielding additional genetic associations which enhance our understanding of the biological mechanisms underlying these complex developmental processes.

## Results

### Genome-wide analysis of HC scores

**Fig. 1:**
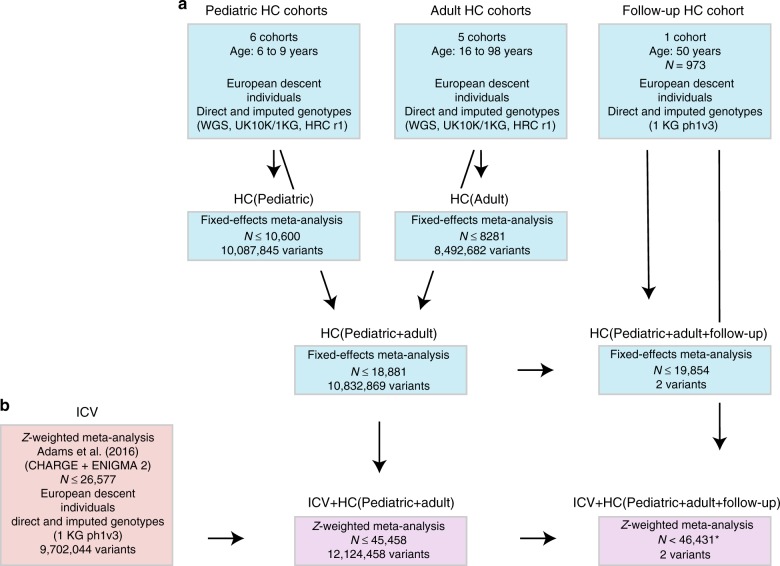
Study design. **a** Head circumference meta-analysis design using a fixed-effect meta-analysis including different developmental stages. **b** Combined head circumference and intracranial volume meta-analysis design using a *Z*-weighted meta-analysis. ICV intracranial volume. WGS whole-genome sequencing; UK10K/1KG Joint UK10K/1000 Genomes imputation template, 1KG 1000 Genomes imputation template, HRC The Haplotype Reference Consortium r1. *Due to sample dropout only *N* ≤ 43,529 were available

**Fig. 2:**
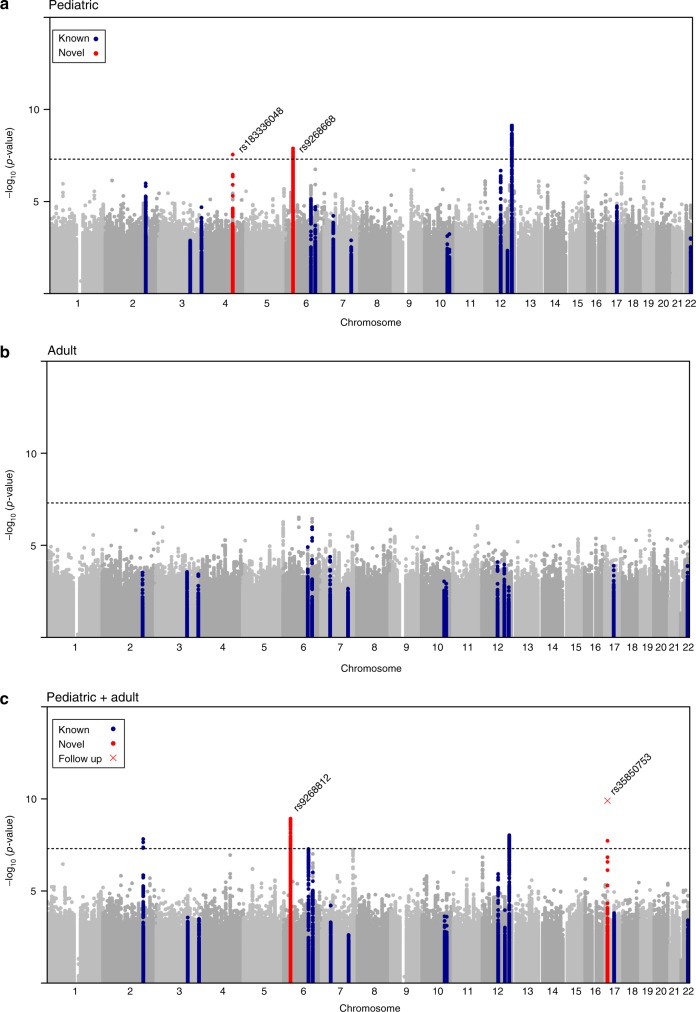
Genome-wide association with final head circumference (HC). **a** HC(Pediatric): *N* = 8281, **b** HC(Adult): *N* = 10,600 and **c** HC(Pediatric + adult): *N* = 18,881 inverse-variance weighted meta-analyses. The dashed line represents the threshold for nominal genome-wide (*p* < 5.0 × 10^−8^) significance. Accounting for multiple testing, the adjusted level of genome-wide significance is *p* < 3.3 × 10^−8^. Known variants for intracranial volume, brain volume, and head circumference are shown in blue. Novel signals passing a nominal genome-wide association threshold (*p* < 5.0 × 10^−8^) are shown with their lead SNP in red. Replicated signals are labeled with a red cross. The genomic position is shown according to NCBI Build 37

**Fig. 3:**
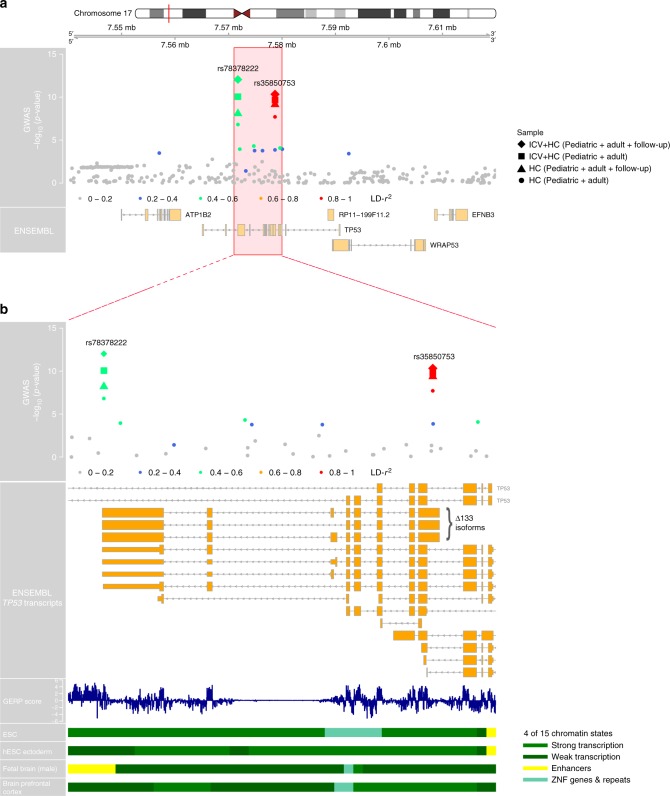
Regional association plot at 17p13.1 associated with final head circumference (HC) and final cranial dimension. **a** Depicts a 800 Mb window and **b** a zoomed view of genetic association signals and functional annotations near *TP53*. Within each plot, in the first panel SNPs are plotted with their −log_10_ *p* value as a function of the genomic position (b37). This panel shows the statistical evidence for association based on HC (Pediatric + adult) and combined ICV + HC (Pediatric + adult) meta-analyses, including HC follow-up studies. SNPs are colored according to their correlation with the HC lead signal (rs35850753, pairwise LD-*r*^2^-values). The second panel represents the gene region (ENSEMBL GRCh37). The third panel in (**b**) presents the Genomic Evolutionary Rate Profiling (GERP++) score of mammalian alignments. The last four panels in (**b**) show 4 of 15 core chromatin states, present in the zoomed view, from the Roadmap Epigenomics Consortium including Embryonic Stem Cells (ESC), hESC Derived CD56 + Ectoderm Cultured Cells, Fetal Brain (Male) and Brain Dorsolateral Prefrontal Cortex respectively (see legend for color coding)

We carried out genome-wide analysis of HC scores using a two-stage developmentally sensitive design (Fig. 1a) including (i) pediatric (6–9 years of age), (ii) adult (16–98 years) and (iii) combined pediatric and adult samples comprising up to 18,881 individuals of European origin from 11 population-based cohorts and 10 million imputed or sequenced genotypes (Supplementary Table 1). Inverse-variance weighted meta-analysis (Supplementary Data 1–4, Fig. 2a–c, Supplementary Figures 1–3) identified three novel regions at chromosome 4q28.1 (HC (Pediatric): lead variant rs183336048, effect allele frequency (EAF) = 0.02, *p* = 3.0 × 10^−8^, Supplementary Figures 5a, 6a), 6p21.32 (HC (Pediatric)/ HC (Pediatric + adult): lead variant rs9268812, EAF = 0.35, *p* = 2.2 × 10^−9^, Supplementary Figures 5b, 6b) and 17p13.1 (Pediatric + adult: lead variant rs35850753, EAF = 0.02, *p* = 2.0 × 10^−8^, Supplementary Figures 5c, 6c, Fig. 3a, b) as associated with HC at an adjusted genome-wide significant level (*p* < 3.3 × 10^−8^) (Table 1). We followed up the two signals in HC (Pediatric + adult) in a further 973 adults of European descent (mean age 50 years) (Supplementary Table 2, Supplementary Figure 6b, c) and replicated directionally consistent evidence for association with rs35850753 at the 17p13.1 locus (*p* = 4.5 × 10^−5^, Table 1). In the combined pediatric, adult and follow-up sample, we observed here an increase of 0.24 sex-adjusted SD units in HC per increase in minor T risk allele (*p* = 2.1 × 10^−10^, Table 1, Fig. 3a, b).

**Table 1:** Novel loci for final head circumference

Variant	Chr:Pos (b37)	EA: EAF	Discovery			Follow-up		Discovery + follow-up		*N*_Total_
			*β* (SE)	*p*	*p*_het_	*β* (SE)	*p*	*β* (SE)	*p*	
HC (Pediatric)										
rs183336048	4: 12830274	T: 0.02	0.31 (0.06)	3.0×10^−8^	0.62	—	—	—	—	10,303
HC (Pediatric + adult)										
rs9268812^a^	6: 32424568	A: 0.35	0.07 (0.01)	1.3×10^−9^	0.85	0.01 (0.05)	0.87	0.07 (0.01)	2.1×10^−9^	19,557
rs35850753	17: 7578671	T: 0.02	0.22 (0.04)	2.0×10^−8^	0.21	0.64 (0.16)	4.5×10^−5^	0.24 (0.04)	2.1×10^−10^	19,557

Genome-wide meta-analysis of head circumference scores in pediatric and adult samples as described in Fig. 1a; Evidence for association was assessed using standard error-weighted fixed effects meta-analysis. Only independent variants (Linkage disequilibrium (LD)-*r*^2^ < 0.2 within ±500 kb) reaching nominal evidence for genome-wide significance (*p* < 5.0×10^−8^) are shown. The adjusted threshold for genome-wide significance is 3.3×10^−8^, accounting for multiple testing. Detailed information can be found in Supplementary Data 1 and 3

*Position* base pair position, *HC* head circumference, *EA* effect allele, *EAF* effect allele frequency, *β* effect estimate, *SE* standard error, *p* *p*-value, *N* sample size, *p*_het_ *p*-value for heterogeneity statistic (based on Cochran’s Q-test for heterogeneity)

^a^rs9268812 is in linkage disequilibrium with rs9268668 (*r*^2^ = 0.60) that passes the adjusted genome-wide significance threshold within the HC(Pediatric) meta-analysis (Supplementary Data 1)

**Fig. 4:**
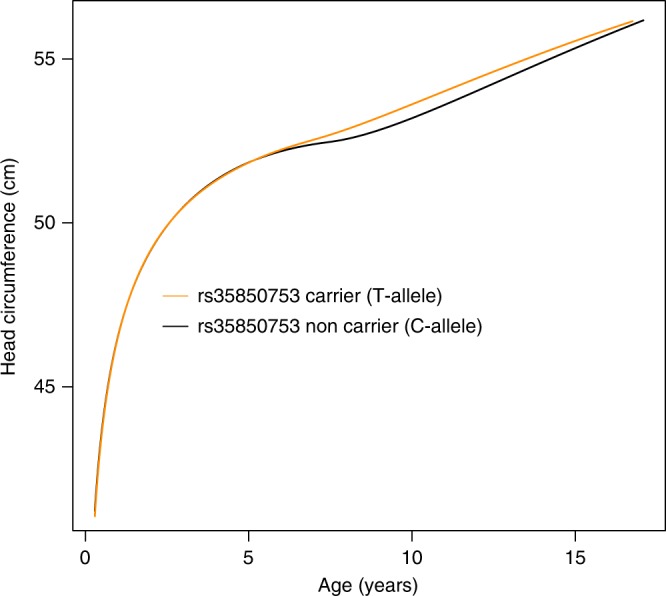
Stratified head circumference growth model trajectories for rs35850753 carriers (T-allele) versus non carriers (C-allele). The growth model was based on untransformed head circumference (cm) scores spanning birth to 15 years observed in 6225 ALSPAC participants with up to 13 repeat measures (17,269 observations) using a mixed effect SuperImposition by Translation And Rotation (SITAR) model

Growth curve modeling of HC scores between birth and the age of 15 years in participants of the ALSPAC sample, using a stratified Super Imposition by Translation And Rotation (SITAR) model^18^, suggested that carriers of the T risk allele at rs35850753 developed larger heads from mid-childhood onwards (Fig. 4), with risk alleles being positively related to individual differences in mean HC (Linear regression, two-sided *p* = 6.9 × 10^−^^12^) and HC growth velocity (Linear regression, two-sided *p* = 7.1 × 10^−11^, Supplementary Table 3). For example, at the age of 10 years male carriers had an HC score of 54.16 cm and noncarriers a score of 53.63 cm. In comparison, female carriers and noncarriers had a score of 53.21 cm and 52.74 cm respectively. rs3585075 resides within the tumor suppressor encoding *TP53* gene and is not related to any known GWAS locus for HC, ICV or brain volume (Supplementary Table 4, Supplementary Figure 4) when conducting a conditional analysis, nor any locus affecting height^19^ (Supplementary Note 2). In addition to these novel associations, our analysis replicated known signals for infant HC on chromosome 12q24.31^13^ and a previously reported joint signal of infant HC and adult ICV on chromosome 2q32.1^12^ (Fig. 2c).

Applying a gene-based test approach^20^, multiple HC-associated genes were identified (Supplementary Data 5–7). The strongest signal in HC (Pediatric), and to a lesser extent in HC (Pediatric + adult), resides at 12q24.31 (lead gene-wide signal *MPHOSPH9*, *p* = 2.3 × 10^−10^) and contains single variants in linkage disequilibrium (LD) with known GWAS signals for infant HC^13^ (e.g. *SBNO1*, *p* = 2.0 × 10^−7^). The strongest gene-wide signals that did not harbor variants in LD with known or novel single GWAS variants were identified at 5q31.3 (Lead gene-wide signal *SLC4A9*, p = 6.6 × 10^−9^), and at 16p13.3 (Lead gene-wide signal *E4F1*, p = 1.6 × 10^−8^), using summary statistics from the HC (Pediatric + adult) meta-analysis. Gene-based analyses were complemented with studies predicting gene expression levels in multiple tissues (Supplementary Table 5). Notably, for the HC (Pediatric) gene-wide signal at 6p21.32, including the *PRRC2A* locus (Gene-wide signal *p* = 7.2 × 10^−7^, Supplementary Data 5), predicted gene expression levels in whole blood were found to be inversely associated with HC scores, using S-PrediXcan^21^ software (*p* = 5.7 × 10^−7^; Supplementary Table 5, Supplementary Data 8).

### Genetic architecture of HC scores during development

**Fig. 5:**
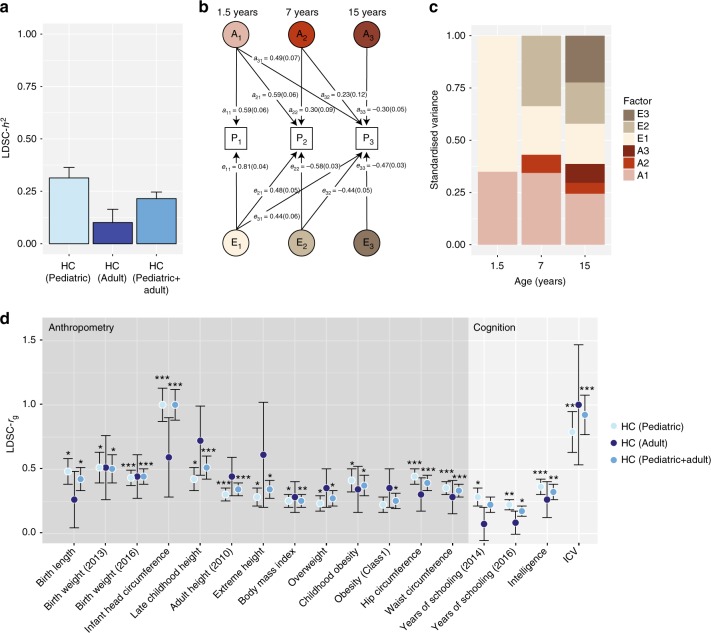
Genetic architecture of head circumference (HC). **a** Linkage-disequilibrium score SNP-heritability (LDSC-*h*^2^) for HC (Pediatric), HC (Adult) and HC (Pediatric + adult) meta-analyses. **b** Genetic-relationship matrix structural equation modeling (GSEM) of head circumference during development: Path diagram of the full Cholesky decomposition model using longitudinal head circumference measures from ALSPAC (1.5 years (*N* = 3945), 7 years (*N* = 5819), and 15 years (*N* = 3406)). Phenotypic variance (P1, P2, P3) was dissected into genetic (A1, A2 and A3) and residual (E1, E2 and E3) factors. Observed measures are represented by squares and latent factors by circles. Single-headed arrows define relationships between variables. The variance of latent variables is constrained to unit variance. **c** Standardized genetic and residual variance components for head circumference during development. Variance components were estimated using the GSEM model as shown in (**b**). **d** Linkage-disequilibrium score correlation (LDSC-*r*_g_) for HC (Pediatric), HC (Adult) and HC (Pediatric + adult) and 235 phenotypes: 17 genetic correlation estimates passing a Bonferroni threshold (*p* < 0.00014) are shown with their standard errors. ***p < 10^−8^; **p < 10^−5^; *p < 0.00014

Linkage-disequilibrium score regression (LDSC)^22^ analyses (Fig. 5a, Supplementary Table 6) using genome-wide summary statistics suggested that heritability estimates during childhood (6−9 years) are higher (SNP-*h*^2^ = 0.31(SE = 0.05)) than in adult samples (16−98 years; SNP-*h*^2^ = 0.097(SE = 0.06)), although 95% confidence intervals marginally overlap. The estimated genetic correlation^23^ between both developmental windows was high (LDSC-*r*_g_ = 1.04(SE = 0.39), *p* = 0.0075). The LD-score regression intercepts were consistent with one for all HC meta-analyses, suggesting little inflationary bias in GWAS (Supplementary Table 6).

To investigate developmental changes in the genetic architecture of HC scores, we carried out a multivariate analysis of genetic variances using genetic-relationship-matrix structural equation modeling (GSEM)^24^. Fitting a saturated Cholesky decomposition model (Fig. 5b) to HC scores assessed in ALSPAC participants (*N* = 7924) at the ages of 1.5, 7, and 15 years (Supplementary Table 7), we observed total SNP-*h*^2^ estimates of 0.35 (SE = 0.07), 0.43 (SE = 0.05), and 0.39 (SE = 0.07) respectively (Fig. 5c). More importantly, this analysis suggested that a large proportion of genetic factors contributing to phenotypic variation in HC scores remains unchanged during the course of development, with genetic factors operating at the age of 1.5 years explaining 63.1% (SE = 9%) and those at age 7 years 76.5% (SE = 5%) of the genetic variance at age 15 years, respectively. Consistently, strong genetic correlations were identified among all scores during development (1.5−7 years, *r*_g_ = 0.89 (SE = 0.07); 1.5−15 years, *r*_g_ = 0.79 (SE = 0.09); 7−15 years, *r*_g_ = 0.87 (SE = 0.04), in support of LD-score correlation analyses.

### Genetic correlation of complex phenotypes with HC

A systematic screen for genetic correlations between HC scores and 235 complex phenotypes using LD score correlation^23^ identified moderate to strong positive genetic correlations (*r*_g_ ≥ 0.3) with many anthropometric and cognitive/cognitive proxy traits. This includes HC scores during infancy, birth weight, birth length, height, extreme height, hip circumference, childhood obesity, waist circumference, intelligence scores, and ICV (Supplementary Table 8, Fig. 5d, Supplementary Data 9). Weaker positive genetic correlations (0 < *r*_g_ < 0.3) were also present for years of schooling, obesity, body mass index, overweight, and extreme height.

The strongest cross-trait genetic correlation was identified between HC (Pediatric + adult) and ICV (*r*_g_ = 0.91(SE = 0.16), *p* = 1.6 × 10^−8^). However, there was little evidence that SNP-*h*^2^ estimates for HC are enriched for genes that are highly expressed in brain tissues or chromatin marks in neural and bone tissue/cell types, beyond chance (Supplementary Data 10) in the conducted HC meta-analyses.

### Combined genome-wide analysis of HC scores and ICV

**Fig. 6:**
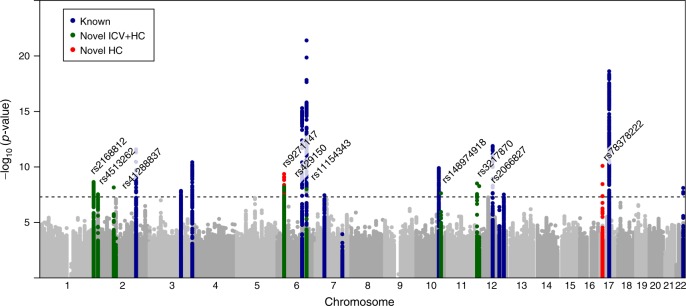
Genome-wide association analysis of final cranial dimension. A genome-wide weighted *Z*-score meta-analysis of combined head circumference (HC) and intracranial volume (ICV) was carried out (ICV + HC (Pediatric + adult): *N* = 45,458). The dashed line represents the threshold for nominal genome-wide (*p* < 5.0 × 10^−8^) significance. Accounting for multiple testing, the adjusted level of genome-wide significance is *p* < 3.3×10^−8^. Known variants for ICV, brain volume, and HC are shown in blue. Novel signals passing a nominal genome-wide association threshold (*p* < 5.0 × 10^−8^) are shown with their lead SNP in green (Table 2, Table S15). HC (Pediatric + adult) signals identified in this study are shown in red. The genomic position is shown according to NCBI Build 37

Given the prior expectation of similar genetic architectures between HC and ICV, supported through genetic correlation analyses, we meta-analyzed both phenotypes by combining HC summary statistics from pediatric and adult cohorts (*N* = 18,881) with ICV summary statistics from the CHARGE and ENIGMA2 consortia^12^ (*N* = 26,577, Fig. 1b) using a *Z*-score weighted meta-analysis (Fig. 6, Table 2, Supplementary Data 11, 12, 13). The strongest evidence for novel genetic association in this combined cranial dimension analysis was observed for the low-frequency marker rs78378222 (MAF = 0.02; *p* = 7.9 × 10^−^^11^) at the 17p13.1 locus, a functional variant that is in LD with rs35850753, the strongest GWAS signal for HC (*r*^2^ = 0.56, *p* = 3.6 × 10^−9^, Fig. 3a, b, Supplementary Data 11). To study the independence of the two signals, we carried out conditional analyses. Adjusting rs35850753 for variation at rs78378222, the association with HC (Pediatric + adult) was strongly attenuated, but remains present at the nominal level (conditional *β* = 0.06 (SE = 0.025), *p* = 0.013). Reciprocally, the signal at rs78378222, conditional on rs35850753, was still detectable at the nominal level in the combined cranial dimension analysis (conditional *β* = 0.051 (SE = 0.017), *p* = 0.0024), based on standardized regression estimates.

**Table 2:** Novel independent loci for combined intracranial volume and head circumference

Variant	Chr:Pos (b37)	EA: EAF	ICV		HC (Pediatric + adult)		ICV + HC (Pediatric + adult)		*N*_Total_
			β* (SE)	*p*	β* (SE)	*p*	β* (SE)	*p*	*N*
rs2168812	1: 243777066	A: 0.18	0.05 (0.01)	9.0×10^−6^	0.06 (0.01)	1.5×10^−5^	0.05 (0.01)	2.3×10^−9^	45,458
rs4513262	2: 10181457	T: 0.21	0.05 (0.01)	7.7×10^−7^	0.04 (0.01)	2.3×10^−3^	0.05 (0.01)	2.9×10^−8^	45,336
rs41288837	2: 85531116	T: 0.03	−0.10 (0.03)	1.0×10^−4^	−0.15 (0.03)	1.6×10^−6^	−0.11 (0.02)	7.2×10^−9^	43,916
rs429150^a^	6: 32075563	T: 0.52	0.04 (0.01)	3.1×10^−5^	0.04 (0.01)	1.0×10^−4^	0.04 (0.01)	3.9×10^−8^	43,122
rs9271147^b^	6: 32577385	T: 0.17	0.06 (0.02)	4.3×10^−4^	0.07 (0.01)	6.5×10^−8^	0.07 (0.01)	4.4×10^−10^	30,057
rs11154343	6: 126412953	T: 0.31	−0.05 (0.01)	1.7×10^−8^	−0.03 (0.01)	1.2×10^−2^	−0.04 (0.01)	1.0×10^−8^	45,458
rs7808664^a^	7: 32917550	A: 0.37	−0.04 (0.01)	1.2×10^−6^	−0.03 (0.01)	2.0×10^−3^	−0.04 (0.01)	3.6×10^−8^	45,458
rs148974918	10: 112033051	T: 0.05	−0.08 (0.02)	9.4×10^−5^	−0.1 (0.02)	1.6×10^−5^	−0.08 (0.02)	2.4×10^−8^	45,458
rs3217870	12: 4400111	T: 0.62	−0.03 (0.01)	1.0×10^−4^	−0.05 (0.01)	7.0×10^−7^	−0.04 (0.01)	3.1×10^−9^	45,458
rs2066827	12: 12871099	T: 0.77	0.05 (0.01)	3.9×10^−6^	0.05 (0.01)	9.3×10^−5^	0.05 (0.01)	5.4×10^−9^	38,871
rs78378222^c^	17: 7571752	G: 0.02	0.15 (0.04)	1.4×10^−5^	0.21 (0.04)	1.5×10^−7^	0.17 (0.03)	7.9×10^−11^	41,371

Genome-wide combined meta-analysis of intracranial volume and head circumference scores as described in Fig. 1b; Evidence for association was assessed using *Z*-weighted meta-analysis. Standardized estimates were derived as described in the Methods. Only independent variants (Linkage disequilibrium (LD)-*r*^2^ < 0.2 within ±500 kb) reaching nominal evidence for genome-wide significance (*p* < 5.0 × 10^−8^) are shown. The adjusted threshold for genome-wide significance is 3.3×10^−8^, accounting for multiple testing. Detailed meta-analysis information can be found in Supplementary Data 11. Detailed SNP and gene information is given in Supplementary Data 16 and 17

ICV—intracranial volume (ICV) meta-analysis of ENIGMA2 and CHARGE samples, *N* ≤ 26,577

HC (Pediatric + adult)—combined pediatric and adult head circumference meta-analysis, *N* ≤ 18,881

*Position* base pair position, *EA* effect allele, *EAF* effect allele frequency, β* standardized effect estimate, *SE* standard error, *p* *p*-value, *N* sample size

^a^Passes a nominal threshold for genome-wide significance only (*p* < 5.0 × 10^−8^)

^b^rs9271147 is in linkage disequilibrium with rs9268812 (*r*^2^ = 0.41), a novel GWAS signal from HC (Pediatric + adult) (Table 1, Supplementary Data 3)

^c^rs78378222 is in linkage disequilibrium with rs35850753 (*r*^2^ = 0.56), a novel GWAS signal from HC (Pediatric + adult) (Table 1, Supplementary Data 3)

In addition, we identified eight independent genetic loci within the combined ICV and HC meta-analysis that have not previously been reported for either HC or ICV (Supplementary Data 11). This includes evidence for association at rs9271147 (MAF = 0.17; *p* = 4.4 × 10^−10^) at 6p21.32 within the MHC region, which is in LD with rs9268812 (*r*^2^ = 0.41), a further signal from our HC (Pediatric + adult) meta-analysis. We also observed increased statistical evidence for association at nine known markers compared to the original studies for either HC, brain volume or ICV (Supplementary Data 12).

Adding further genotype information from the HC follow-up cohort (*N* = 973, Fig. 1b), strengthened the evidence for association at both rs78378222 and rs35850753 (*p* = 8.8 × 10^−13^ and *p* = 4.9 × 10^−11^ respectively, total *N* ≤ 43,529, Fig. 3a, b, Supplementary Data 11), corresponding to a change in 0.19 (SE = 0.03) and 0.16 (SE = 0.02) standard deviation (SD) units respectively. However, within neuroimaging samples only, support for association at rs78378222 was low in the combined CHARGE and ENIGMA2 samples^12^ (Table 2).

Note that genetic effects with respect to a combined final cranial dimension cannot be translated into absolute units as HC scores and ICV relate to diametric versus volumetric properties respectively.

### Biological and phenotypic characterization of signals

A detailed variant annotation of all novel signals for the combined ICV and HC meta-analysis, including overlapping signals from the HC meta-analysis alone, was carried out using the FUMA webtool^25^ (Supplementary Data 14−18) including variant annotation (Supplementary Data 16), mapped genes (Supplementary Data 17), and previously published studies (Supplementary Data 18).

The strongest cranio-dimensional signal, rs78378222, resides within the 3′ untranslated region (UTR) of *TP53* and the low-frequency allele leads to a change in the *TP53* polyadenylation signal that results in impaired 3′-end processing for many *TP53* mRNA. The strongest HC signal, rs35850753, resides within the 5′-UTR of the Δ133 *TP53* isoforms and otherwise intronically (Fig. 3b, Supplementary Data 16). Species comparison showed that variation at rs78378222 is highly conserved (GERP-score = 5.28)^26^ and also predicted to be deleterious (CADD = 17.97)^27^, while variation at rs35850753 is not (GERP-score = −2.8; CADD = 1.04) (Fig. 3b, Supplementary Data 16). According to a core 15-state chromatin model, variation at rs78378222, but not at rs35850753, is furthermore in LD (*r*^2^ = 0.8) with an enhancer in fetal brain (Fig. 3b). Using the FUMA webtool^25^ and Brain xQTL^28^, we found no support for blood or brain cis eQTLs or meQTL in LD (*r*^2^ > 0.6) with either rs35850753 or rs78378222, when adjusted for the number of loci tested (Supplementary Data 19). The strongest evidence for cis eQTL at *TP53* was found for eQTL in modest LD with rs35850753 (*r*^2^ = 0.22), explaining variation in gene level *TP53* transcript in blood, with the rare T risk allele being associated with lower full-length transcript levels (False Discovery Rate *q* = 6 × 10^−6^, Supplementary Data 17). We also characterized the *TP53* association signals for final HC and combined final ICV + HC phenotypically using a phenome-wide scan in the UK Biobank, as implemented in PHESANT^29^ (rs35850753: Supplementary Data 20). For rs35850753, standing height is increased by 0.012 cm (SE = 0.002) and sitting height by 0.015 cm (SE = 0.003) for each increase in minor effect allele. The log odds of having an inpatient primary diagnosis code for “Fracture of tooth” increase by 0.42 (SE = 0.078) and the log odds of a participant answering “yes” to “ever had hysterectomy” by 0.06 (SE = 0.011) per effect allele. For each increase in minor effect allele at rs78378222, standing height is increased by 0.015 cm (SE = 0.002) and sitting height by 0.019 cm (SE = 0.003). The log odds of a participant answering “yes” to “ever had hysterectomy” was 0.065 (SE = 0.011) per effect allele. Sensitivity analysis adjusting, in addition, for ten principal components did not change the nature of these findings (Supplementary Data 20).

Furthermore, we identified variants in LD with the two cranial dimension signals at 1q44 and at 2p25.1 respectively that are predicted to be deleterious. rs12408455 (*r*^2^ with rs2168812 = 0.99) is an intronic SNP within *AKT*3 (CADD = 17.87) and rs112040334 (*r*^2^ with rs4513262 = 0.91) an intronic 11-bp insertion/deletion within *KLF11* (CADD = 17.22, Supplementary Data 16). Both variants are in LD (*r*^2^ > 0.6) with eQTL in blood (Supplementary Data 17), with the effect allele at both variants being associated with increasing transcript levels of *AKT3* and *KLF11* respectively. The variant with the highest CADD score identified in the combined ICV and HC meta-analysis is rs41288837. This low-frequency missense variant at 2p11.2 within *TCF7L1* is predicted to belong to the 0.5% most deleterious substitutions in the human genome (MAF = 0.03, CADD = 24.30, Table 2, Supplementary Data 16). We observed no evidence for associated eQTL in blood or brain for this variant (Supplementary Data 17).

Notably, many genes containing SNPs in LD (*r*^2^ > 0.6) with lead variants from the combined ICV and HC meta-analysis show mapped chromatin interactions within mesenchymal and human embryonic stem cells and mesoendoderm (Supplementary Data 17), including *TP53*.

## Discussion

Investigating up to 46,000 individuals of European descent, this study identifies and replicates evidence for genetic association between a novel region on chromosome 17p13.1 and both final HC and ICV + HC, implicating low-frequency variants of large effect within *TP53*. We furthermore demonstrate that the genetic architecture of HC is developmentally stable and genetically correlated with ICV. This is supported by the identification of eight further common and rare independent loci that are associated with cranial dimension as a combined HC and ICV phenotype, illustrating the allele frequency spectrum of the underlying genetic architecture.

For the final HC, the strongest evidence for association at 17p13.1 is observed with rs35850753, while final cranial dimension is most strongly associated with rs78378222. Both rs35850753 and rs78378222 are low-frequency variants, in partial linkage disequilibrium, and their effect sizes are substantially larger than any previously reported GWAS signals for either HC or ICV alone, reaching nearly a fifth and quarter of a SD unit change in final cranial dimension and final HC per rare effect allele respectively. For HC, this translates into an increase of approximately 0.5 cm in HC between carriers and noncarriers of rare alleles at the age of 10 years. Based on longitudinal analyses, it is most likely that genetic effects of rs35850753 on final HC start to emerge during mid-childhood, while we have no comparable longitudinal data source available to evaluate trajectory effects on cranial dimension.

*TP53* encodes the p53 protein, a transcription factor that binds directly and specifically as a tetramer to DNA in a tissue- and cell-specific manner and has a range of antiproliferative functions, lending it the nickname guardian of the genome. The activation of p53 in response to cellular stress promotes cell cycle arrest, DNA repair, and apoptosis^30^. *TP53* mutations are present in approximately 30% of tumor samples making it one of the most studied genomic loci with over 27,000 somatic and 550 germline mutations described to date (Source—IARC TP53 database^31^). The low-frequency allele at rs78378222 leads to a change in the *TP53* polyadenylation signal that results in impaired 3′-end processing and termination of many *TP53* mRNA isoforms^32^, including full-length *TP53* isoforms, although rs78378222 is also in LD with an enhancer region in fetal brain. In contrast, rs35850753 resides in the 5′ UTR of *TP53 Δ133* isoforms that are transcribed by an alternative promoter. This leads to the expression of an N-terminally truncated p53 protein, initiated at codon 133, lacking the trans activation domain^33^. *TP53 Δ133* isoforms are known to directly and indirectly modulate p53 activity and differentially regulate cell proliferation, replicative cellular senescence, cell cycle arrest and apoptosis in response to stress such as DNA damage, including the inhibition of tumor suppressive functions of full-length p53. This mechanism is consistent with the observed link between the rs35850753 low-frequency T allele and lower full-length *TP53* transcript level. Thus, rare effect alleles at both rs78378222 and rs35850753 could potentially, via different biological mechanisms, be linked to impaired p53 activity and thus heightened proliferative potential and less apoptosis of normal human cells, consistent with larger HC scores and a larger cranial dimension.

It is noteworthy that rs78378222 has been previously associated to risk of cancers including tumors of the nervous system such as glioma^34^, a malignant tumor of glial tissue, possibly including neural stem cells, glial progenitors and astrocytes as cells of origin^35^, but also prostate cancer and colorectal adenoma^32^. Both rs35850753 and rs78378222 have also been robustly associated with neuroblastoma^36^, a sympaticoadrenal lineage neural crest-derived tumor. Evidence for neurological phenotypic consequences of *TP53* variation has recently been strengthened by the discovery of *TP53* as a risk locus for general cognitive function using a gene-based approach^37^. *TP53* knockout mouse embryos show furthermore broad cranial defects involving skeletal, neural, and muscle tissues^38^. Similarly, mouse models for Treacher Collins syndrome (a disorder of cranial morphology which arises during early embryological development as a result of defects in the formation and proliferation of neural crest cells) could be rescued by inhibition of p53 during embryological patterning^39^. In particular, there is support from animal and tissue models for a role of p53 in neural crest cell (NCC) development^38^ with NCCs supplementing head mesenchyme during fetal development^11,40^. NCCs also contribute to the development of a thick three-membrane layer called the meninges, that cover the telencephalon^11,40^ and directly locate underneath the skull. In particular, Pia mater, the innermost layer of the meninges, adheres closely to sulci and fissures of the cortex. During postnatal brain growth, including extensive increases in myelinated white matter^41^, the calvarial bones are drawn outward, partially due to the expanding meninges, triggering the production of membranous skull bone^40^. Moreover, meninges have been thought to play a key role in the coordinated integration of signaling pathways regulating both neural and skeletal cranial growth^11^. It is possible to speculate that p53 is part of these joint regulatory mechanisms, for example, via Wnt signaling regulation^42^. However, beneficial effects of rs35850753 and rs78378222 on growth patterning leading to an increased HC and cranial dimension might be counterbalanced by adverse outcomes such as glioblastoma, keeping both variants at a lower frequency.

Combined analysis of HC and ICV, as related measures of final cranial dimension, also identified association at eight further loci, in addition to variation at 17p13.1 and loci previously reported for either infant HC and/or adult ICV. This includes the low-frequency variant rs41288837, predicted to belong to the 0.5% most deleterious substitutions in the human genome. The variant exerts moderately large effects that correspond to an approximately 10% decrease in SD units of final cranial dimension per rare T allele. rs41288837 is a missense variant in *TCF7L1* at 2p11.2, a locus encoding a transcription factor mediating Wnt signaling pathways that are known to play an important role in vertebrate neural development^43^. The effects of this variant were consistent for both HC and ICV, although each of the individual trait analyses was too underpowered to detect association at this variant at a genome-wide level. An additional low-frequency variant in this study, rs183336048 at 4q28.1, was identified as associated with pediatric HC only, but could not be replicated due to a lack of comparable age-matched follow-up cohorts. rs183336048 lies 5′ to *INTU* which encodes inturned planar cell polarity protein, a polarity effector affecting neural tube patterning and cilliation^44^. Common variants identified in the ICV + HC combined meta-analysis also include intronic variation in *AKT3* (rs2168812) and *CCND2* (rs3217870), which are related through the phosphatidylinositol 3-kinase (PI3K-AKT) pathway^45^. Disruption of PI3K-AKT pathway components causes megalencephaly-polymicrogyria-polydactyly-hydrocephalus syndrome and a spectrum of related megalencephaly syndromes^45,46^. This supports previous in silico pathway analysis, which nominated PI3K-AKT, an intracellular signaling pathway controlling the cell cycle, as candidate pathway for intracranial volume^12^. Both, rs2168812 and rs3217870, have also been associated with cancer risk, similarly to the two *TP53* variants, with the *AKT3* variant rs12076373 (LD*r*^2^ with rs2168812 = 0.70) being related to risk for non-glioblastoma brain tumors^34^ and the *CCND2* variant rs3217901 (LD*r*^2^ with rs3217870 = 0.63) being related to colorectal cancer (Supplementary Data 18). Notably, AKT3 signaling is an essential intracellular pathway controlling neural crest development^47^, while tissue-specific chromatin interactions in mesenchymal stem cells have been reported for several novel loci (Supplementary Data 17), supporting a role of neural crest-related processes in shaping final cranial dimension.

Genetic correlation analyses provided strong evidence for shared genetic determinants between HC and both anthropometric (birth weight, height, waist and hip circumference) and cognitive traits, as well as ICV. Genetic correlations with waist circumference and hip circumference recapitulate observed correlations between the size of the maternal pelvis and the size of the neonatal cranium^48^, possibly induced because bipedal locomotion limits pelvic size. This is important as a mismatch between the maternal pelvis and the fetal head^49^, i.e. a cephalopelvic disproportion (CPD, also known as fetopelvic disproportion), can put the lives of both mother and fetus at risk, if left untreated. Mathematical models show that evolutionary forces such as a weak directional selection for a large neonate and/or a weak selection for a narrow pelvis can account for the considerable incidence of CPD in humans^50^, and predict a further rise in CPD incidence due to the frequent use of Caesarian sections during recent years.

We also confirmed previously reported genetic links between educational attainment and infant HC^51^, and identified additional evidence for genetic correlation between HC and intelligence, especially pediatric HC. With strongly shared genetic liability (i.e. a genetic correlation coefficient near one), we considered HC and ICV to be related proxy measures of an underlying phenotype, which we termed final cranial dimension. This also suggests that estimated skeletal volume, a combination of HC, cranial height and cranial length^52^, might represent a more accurate, easily accessible and inexpensive measure to enhance power for future genetic analysis using a multi-trait approach^53^ in combination with ICV, exploiting similar volumetric properties.

Multivariate analyses of genetic variance showed that genetic factors contributing to variation in HC during infancy explain the majority of genetic variance during later life, although novel genetic influences arise both during mid-childhood and adolescence. This is further reflected in strong GSEM-based genetic correlations across childhood and adolescence, and strong LDSC-based genetic correlations between infant and adult HC. The estimated LDSC-*h*^2^ of HC in adult samples was lower than in pediatric samples, with only marginally overlapping 95% confidence intervals, implying that phenotypic variation in final HC is less well accounted for by genetic influences than variation in childhood HC, probably as skeletal growth processes have ceased.

The discovery that low-frequency variation, especially near *TP53*, is associated with HC demonstrates the scientific value of testing for variation in the lower allele frequency spectrum and the utility of comprehensive imputation templates. Low-frequency variants identified in this study had larger effects than common variants (Supplementary Figures 7 and 8), in keeping with findings from a range of complex phenotypes including anthropometric traits^17,54,55^. Nevertheless, despite having sufficient power to detect low-frequency variation explaining as little as 0.11% of the variance in HC, this study was underpowered for rare variant analysis (Supplementary Note 4), underlining the need for even larger research efforts. Collectively, our findings provide insight into the genetic architecture of cranial development and contribute to an improved understanding of its dynamic nature throughout human growth and development.

## Methods

### Study population

For the discovery analysis, we adopted a two-stage developmental design including cohorts with HC scores during childhood (Pediatric HC, mean age 6−9 years of age, *N* = 10,600), during adulthood (Adult HC, mean age 44−61 years of age, *N* = 8281) and a combination thereof (*N* = 18,881) including individuals of European descent from 11 population-based cohorts (Supplementary Tables 1 and 2, Fig. 1). Cohorts include The Avon Longitudinal Study of Parents and Children (ALSPAC), the Generation R Study (GenR), the Western Australia Pregnancy Cohort Study (RAINE), the Copenhagen Prospective Study on Asthma in Children (COPSAC2000 and COPSAC2010), the Infancia y Medio Ambiente cohort (INMA), the Hellenic Isolated Cohorts HELIC-Pomak and HELIC-MANOLIS, the Orkney Complex Disease Study (ORCADES), the Croatian Biobank Korčula (CROATIA-KORCULA), and the Viking Health Study-Shetland (VIKING). Within ALSPAC analysis was performed separately in individuals with whole-genome sequence data (ALSPAC WGS) and chip-based genotyping (ALSPAC GWA). For follow-up, we studied 973 individuals from the Croatian Biobank, Split (CROATIA-SPLIT) (Supplementary Table 2). Institutional and/or local ethics committee approval was obtained for each study. Written informed consent was received from every participant within each cohort, and this study has complied with all ethical regulations. An overview of each cohort can be found in Supplementary Tables 1 and 2 with more detailed information in Supplementary Note 1.

### Genotyping

Within ALSPAC, we obtained low read depth (average  × 7) whole-genome sequencing data (ALSPAC WGS)^55^. Chip-based genotyping was performed on various commercial genotyping platforms, depending on the cohort (Supplementary Table 1). Prior to the imputation, all cohorts had similar quality control; variants were excluded because of high levels of missingness (SNP call rate < 98%), strong departures from Hardy−Weinberg equilibrium (*p* < 1.0 × 10^−6^), or low MAF (<1%). Individuals were removed if there were sex discordance, high heterozygosity, low call rate (<97.5%) or duplicates. For imputation, the reference panel was either joint UK10K/1000 Genomes^55^ or the Haplotype Reference Consortium^15^. Additional details can be found in Supplementary Table 1 and Supplementary Note 1.

In addition to study-specific quality control measures, central quality control was performed using the EasyQC R package^56^. First, variants were filtered for imputation quality score (imputed studies only, INFO > 0.6), minor allele count (MAC; ALSPAC WGS MAC > 4, all imputed studies MAC > 10) and a minimum MAF of 0.0025. SNPs with MAF discrepancies (>0.30) compared to the HRC panel were also excluded. Marker names were harmonized and reported effect and noneffect alleles were compared against reference data (Build 37). Variants with missing or mismatched alleles were dropped, in addition all insertion/deletions (INDELs), duplicate SNPs and multiallelic SNPs were excluded. The reported EAF for each study was plotted against the frequency in the HRC reference data to identify possible strand alignment issues (Supplementary Figures 1, 3). The final number of variants passing all quality control tests and the per-study genomic inflation factor (*λ*) are reported in Supplementary Tables 1 and 2.

### Phenotype preparation

Pertinent to this study, HC measures in all individual cohorts were transformed into *Z*-scores using a unified protocol. After the removal of outliers (±4 SD within each sample), HC was adjusted for age within males and females separately. Residuals for each sex were subsequently transformed into *Z*-scores and eventually combined (thus removing inherent sex-specific effects). Note that the phenotype transformation within ALSPAC was jointly carried out for both sequenced and genome-wide imputed samples.

### Genetic-relationship structural equation modeling

Developmental changes in the genetic architecture of HC scores between the ages of 1.5 and 15 years were modeled using genetic-relationship structural equation modeling (GSEM, R gsem library, v0.1.2)^24^. This multivariate analysis of genetic variance combines whole-genome genotyping information with structural equation modeling techniques using a full information maximum likelihood approach^24^. Changes in genetic variance composition were assessed with longitudinal HC scores in ALSPAC participants (7924 individuals with up to three measures; 1.5 years, *N* = 3945; 7 years *N* = 5819; 15 years, *N* = 3406). HC scores were *Z*-standardized at each age, as described above. Genetic-relationship matrices were constructed based on directly genotyped variants in unrelated individuals, using GCTA software^57^, and the phenotypic variance dissected into genetic and residual influences using a full Cholesky decomposition model^24^.

### Multiple testing correction

Using Matrix Spectral Decomposition (matSpD)^58^, we estimated that we analyzed 1.52 effective independent phenotypes within this study (Pediatric, Adult and Pediatric + adult HC scores and ICV^12^ scores) according to the LDSC-based genetic correlations^22^.

### Single variant association analysis

Single variant genome-wide association analysis, assuming an additive genetic model, was carried out independently within each cohort using standard software (Supplementary Table 1, Supplementary Note 1). Residualized HC scores (*Z*-scores) were regressed on genotype dosage using a linear regression framework. For cohorts with unrelated subjects (Supplementary Table 1) association analysis was carried out using SNPTEST v2.5.0 (-method expected, -frequentist)^59^. Note that HC scores in GenR were, in addition, adjusted for four principal components. Cohorts with related participants (HELIC cohorts) utilized a linear mixed model to control for family and cryptic relatedness, implemented in GEMMA^60^.

Individual cohort level summary statistics for HC were combined genome-wide with standard error-weighted fixed effects meta-analysis, allowing for the existence of age-specific effects through an age-stratified design (Fig. 1a). We restricted each HC meta-analysis (Pediatric, Adult, Pediatric + adult) to variants with a minimum sample size of *N* > 5000. Genomic control correction was applied at the individual cohort level and heterogeneity between effects estimates was quantified using the *I*-squared statistic as implemented in METAL^61^. Accounting for the effective number of independent phenotypes studied, the threshold for genome-wide significance was fixed at 3.3 × 10^−8^ and the threshold for suggestive evidence at 6.6 × 10^−6^.

We contacted all studies (known to us) with (a) HC information available in later childhood or adult samples, (b) participants of European ancestry and (c) genotype data. Studies with whole-genome sequencing or densely imputed genotype data (HRC or UK10K/1KG combined templates) were included in the HC meta-analysis, while studies with imputation to other templates were reserved for follow-up. Following this strategy, the majority of studies were included in the meta-analysis, with follow-up in a single study.

### Identification of known variants and conditional analysis

Known GWAS signals (*p* *≤* 5.0 × 10^−8^) were identified from previous studies on HC in infancy^13^, ICV^12,62–64^ and brain volume^65^ using either published or publicly available data (Supplementary Table 4). Conditional analysis was performed with GCTA software using summary statistics from HC (Pediatric) and HC (Pediatric + adult) meta-analyses (Supplementary Figure 4). In addition, we carried out an LD clustering of independent signals from the HC (Pediatric + adult) meta-analysis with respect to all known loci. Briefly, LD clustering is an iterative process that starts with the most significant SNP, which is clumped with variants that have pairwise LD of *r*^2^ ≥ 0.2 within 500 kb using PLINK v1.90b3w, and all variants in LD are removed. Then, the same clumping procedure is repeated for the next top SNP and the iteration continues until there are no more top variants with *p* < 1.0 × 10^−4^. For details, see Supplementary Note 2. For sensitivity analysis, we repeated the LD clustering with known loci for height as identified through the GIANT consortium (697 known independent height GWAS signals^19^, *r*^2^ = 0.2, ±500 kb).

### Combined meta-analysis of HC and ICV

We carried out a weighted *Z*-score meta-analysis of the combined HC (Pediatric + adult) meta-analysis and the largest publicly available genome-wide summary statistics on intracranial volume (ICV; *N* = 26,577) based on data from Cohorts for Heart and Aging Research in Genomic Epidemiology (CHARGE) and the Enhancing NeuroImaging Genetics through Meta-Analysis (ENIGMA) consortium^12^. A weighted *Z*-score meta-analysis was carried out using METAL^61^ using standardized regression coefficients and 12,124,458 imputed or genotyped variants, assuming a genome-wide threshold of significance at *p* *≤* 3.3 × 10^−8^.

We used the *Z*-scores (*Z*) from the METAL output to calculate the standardized regression coefficient (*β*) for each SNP and trait^66^

1 $$\widehat {\beta _j} \approx Z_j\frac{{\widehat {\sigma _y}}}{{\sqrt {N_j \times 2(1 - {\mathrm {EAF}}_j){\mathrm {EAF}}_j} }},$$

where SNP_*j*_ has an effect allele frequency (EAF_*j*_) and $\widehat {\sigma _y}$ is standard deviation of the phenotype, which is assumed to equal one for standardized traits. The standard error (SE) is calculated as

2 $$Z_j = \frac{{\widehat {\beta _j}}}{{{\mathrm {SE}}(\widehat {\beta _j})}}.$$

To disentangle lead signals observed in both the HC (Pediatric + adult) and the combined ICV + HC (Pediatric + adult) meta-analysis, variants were conditioned on each other using GCTA software and summary statistics.

### Gene-based analysis

Gene-based tests for association were performed using MAGMA^20^, which calculates gene-based test statistics from SNP-based test statistics, position-based gene annotations and a linkage disequilibrium reference panel of UK10K haplotypes using an adaptive permutation procedure. SNP-based test statistics were annotated using mapping files with a 50 kb symmetrical window around genes. For gene definition, we used all 19,151 protein-coding gene annotations from NCBI 37.3 and corrected for the number of genes and effective phenotypes tested, using an adjusted Bonferroni threshold of 1.7 × 10^−6^.

### S-PrediXcan

We used the S-PrediXcan method^21^ as a summary-statistic-based implementation of PrediXcan to test for association between tissue-specific imputed gene expression levels and HC, implemented in the MetaXcan standalone software (v0.3.5). This approach first predicts the transcriptome level using publicly available transcriptome datasets. Then, it infers the association between gene and phenotype of interest, by using the SNP-based prediction of gene expression as weights (predicted from the previous step) and combines it with evidence for SNP association based on phenotype-specific GWAS summary statistics. We predicted gene expression levels for cerebellum (4778 genes; GTEx v6p; Supplementary Note 3), cortex (3177 genes; GTEx v6p; Supplementary Note 3), and whole blood (6669 genes; DGN; Supplementary Note 3) using an adjusted Bonferroni threshold of *p* < 2.3 × 10^−6^ across all tissues tested.

### Estimation of heritability and genetic correlation

Linkage-disequilibrium score regression (LDSC)^22^ was carried out to estimate the joint contribution of genetic variants as tagged by common variants (SNP-*h*^2^) to phenotypic variation in HC. The method is based on GWAS summary statistics and exploits LD patterns in the genome and can distinguish confounding from polygenic influences^22^. To estimate LDSC-*h*^2^, genome-wide *χ*^2^-statistics are regressed on the extent of genetic variation tagged by each SNP (LD-score). The intercept of this regression minus one estimates the contribution of confounding bias to inflation in the mean *χ*^2^-statistic. LD score regression was performed with LDSC software (v1.0.0) and based on the set of well-imputed HapMap3 SNPs (~1,145,000 SNPs with MAF > 5% and high imputation quality such as an INFO score of 0.9 or higher) and a European reference panel of LD-scores. *LD-score correlation* analysis can be used to estimate the genetic correlation (*r*_g_) between distinct samples by regressing the product of test statistics against the same LD-score^23^. Bivariate LD score correlation was performed with the LDHub platform^67^ v1.9.0 (Supplementary Note 5). We assessed the genetic correlation between HC scores and a series of 235 phenotypes (excluding UK Biobank) comprising anthropometric, cognitive, structural neuroimaging and other traits as described in Zheng et al.^67^, with an adjusted Bonferroni threshold of *p* < 1.4 × 10^−4^.

### Stratified LD score regression

Stratified LD score regression^68^ is a method for partitioning heritability from GWAS summary statistics with respect to genes that are expressed in specific tissue/cell types. We applied this method to HC summary statistics to evaluate whether the heritability of HC is enriched for genes that are highly expressed in brain tissues. GTEx v6p (Supplementary Note 3) provided gene expression data from 13 brain tissue/cell types. Each of these tissue annotations was added to the baseline model and enrichment was calculated with respect to 53 functional categories. This is for each functional category the proportion of SNP-*h*^2^ divided by the proportion of SNPs in that category. We performed stratified LD score regression with independent data from the Roadmap Epigenomics consortium and ENCODE project (Supplementary Note 3), where we restricted the analysis to 55 chromatin marks identified in neural and bone tissue/cell types. Similar to the deriving enrichment in gene expression, each annotation was added to the baseline model. Chromatin analysis includes the union and the average of cell-type-specific annotations within each mark. In the joint gene expression and chromatin enrichment analysis, we applied a multiple testing of *p* < 4.8 × 10^−4^ accounting for 68 neural and bone tissues/cell types tested (data from GTEx v6p, ENCODE and Roadmap; Supplementary Note 3).

### Functional annotation of novel signals

Functional consequences of novel variants were explored using two web-based tools: Brain xQTL^28^ and FUMA (v1.3.1)^25^. The threshold for multiple testing for eQTL was adjusted according to the number of genes near the studied novel signals and their proxy SNPs (*r*^2^ = 0.2 and ±500 kb). For Brain xQTL, we corrected for multiple testing based on a threshold of *p* < 7.4 × 10^−4^ to account for 68 genes tested. For FUMA eQTL analysis, a multiple testing threshold of *p* < 7.6 × 10^−4^ was applied to adjust for 42 genes and, 24 blood and brain tissues/cell types (Supplementary Note 3).

### UK Biobank phenome scan

To characterize the phenotypic spectrum of identified HC signals, we conducted a phenome scan on 2143 phenotypes in the UK Biobank cohort^69^, using PHESANT^29^ software (v0.13). Analyses were restricted to participants of UK ancestry (UK Biobank specified variable). One from each pair of related individuals, individuals with high missingness, heterozygosity, gender mismatch and putative aneuploidies were excluded. Genotype dosage at lead single variants identified with GWAS was converted into best-guess genotypes using PLINK v1.90b3w. Linear, ordinal logistic, multinomial logistic and logistic regressions were fitted to test the association between genotype and continuous, ordered categorical, unordered categorical and binary outcomes respectively. Analyses were adjusted for age, sex and genotyping chip, and, for sensitivity analysis, 10 principal components. A conservative Bonferroni threshold was applied accounting for a total of 11,056 tests performed and two genotypes tested (*p* < 2.26 × 10^−6^).

### HC growth curve modeling

Trajectories of untransformed HC (cm) spanning birth to 15 years were modeled in 6225 ALSPAC participants with up to 13 repeat measures (17,269 observations) using a mixed effect SITAR model^18^ (R sitar library v1.0.11). SITAR comprises a shape invariant mixed model with a single fitted curve, where individual curves are matched to the mean curve by modeling differences in mean HC, differences in timing of the pubertal growth spurt and differences in growth velocity^18^. Individuals with large measurement errors, i.e. with HC scores at younger ages exceeding scores at later ages (by more than 0.5 SD of the grand mean) as well as outliers (with residuals outside the 99.9% confidence interval) were excluded. The best fitting model was identified using likelihood ratio tests and the Bayesian Information Criterion and included four fixed effects for splines, a fixed effect for differences in mean HC and a fixed effect for sex, in addition to two random effects for differences in mean HC and growth velocity. Stratified models were fitted for carriers and noncarriers of increaser-alleles at candidate loci. To examine the relationship between genotype dosage and differences in HC and growth velocity, these random effects were regressed on genotype dosage using a linear model.

## Supplementary Information

Supplementary Information

Peer Review Files

Description of Additional Supplementary Files

Supplementary Data 1

Supplementary Data 2

Supplementary Data 3

Supplementary Data 4

Supplementary Data 5

Supplementary Data 6

Supplementary Data 7

Supplementary Data 8

Supplementary Data 9

Supplementary Data 10

Supplementary Data 11

Supplementary Data 12

Supplementary Data 13

Supplementary Data 14

Supplementary Data 15

Supplementary Data 16

Supplementary Data 17

Supplementary Data 18

Supplementary Data 19

Supplementary Data 20

## Data Availability

Genome-wide summary statistics and further analyses in this work that support the findings of this study have been deposited at “The Language Archive”, a public data archive hosted by the Max Planck Institute for Psycholinguistics. Data are accessible with a persistent identifier (https://hdl.handle.net/1839/ff12326d-9688-4a46-bc2a-b50cbbd2b20c). The content can also be found through the Data Archiving and Networked Services database, the Dutch national organization for sustained access to digital research data. All Supplementary Data files can be found under the following links: Supplementary Data 1 Supplementary Data 2 Supplementary Data 3 Supplementary Data 4 Supplementary Data 5 Supplementary Data 6 Supplementary Data 7 Supplementary Data 8 Supplementary Data 9 Supplementary Data 10 Supplementary Data 11 Supplementary Data 12 Supplementary Data 13 Supplementary Data 14 Supplementary Data 15 Supplementary Data 16 Supplementary Data 17 Supplementary Data 18 Supplementary Data 19 Supplementary Data 20
