# Supplementary material for: Low-frequency variation in TP53 has large effects on head circumference and intracranial volume
Source: Nat Commun. 2019 Jan 21;10:357. doi: 10.1038/s41467-018-07863-x (PMC6341110; doi:10.1038/s41467-018-07863-x)
Supplement: Supplementary file 1 — Supplementary Information [file 41467_2018_7863_MOESM1_ESM.pdf]

Supplementary Information for

Low frequency variation in *TP53* has large effects on head circumference and intracranial volume

Haworth *et al*

Table of Contents

Supplementary Figures
Supplementary Figure 1: Allele frequency plots for all genotyping studies in the HC analysis
Supplementary Figure 2: QQ plots for all genotyping studies in the HC analysis
Supplementary Figure 3: Allele frequency and QQ plots for ALSPAC-WGS in the HC analysis
Supplementary Figure 4: QQ plots for conditional and unconditional HC meta-analyses
Supplementary Figure 5: Regional plots for novel HC signals
Supplementary Figure 6: Forest plots for novel HC signals
Supplementary Figure 7: Allele frequency spectrum of associated loci in the HC analysis
Supplementary Figure 8: Allele frequency spectrum of associated loci in the combined ICV+HC analysis

Supplementary Tables
Supplementary Table 1: HC cohort information for genome-wide analysis discovery samples
Supplementary Table 2: HC cohort information for the follow-up sample (Adult)
Supplementary Table 3: Association between rs35850753 and HC growth
Supplementary Table 4: Known signals for HC and ICV
Supplementary Table 5: Predicted gene expression for HC
Supplementary Table 6: Unconstrained LD-score regression analysis
Supplementary Table 7: Genetic-relationship-matrix structural equation modelling of HC
Supplementary Table 8: Unconstrained LD-score genetic correlation analysis

Supplementary Notes
Supplementary Note 1: Cohort descriptions
Supplementary Note 2: Generating lead SNPs
Supplementary Note 3: Gene expression and epigenetic resources
Supplementary Note 4: Power analysis
Supplementary Note 5: Acknowledgments
Supplementary Note 6: Web resources

Supplementary References

Supplementary Figures

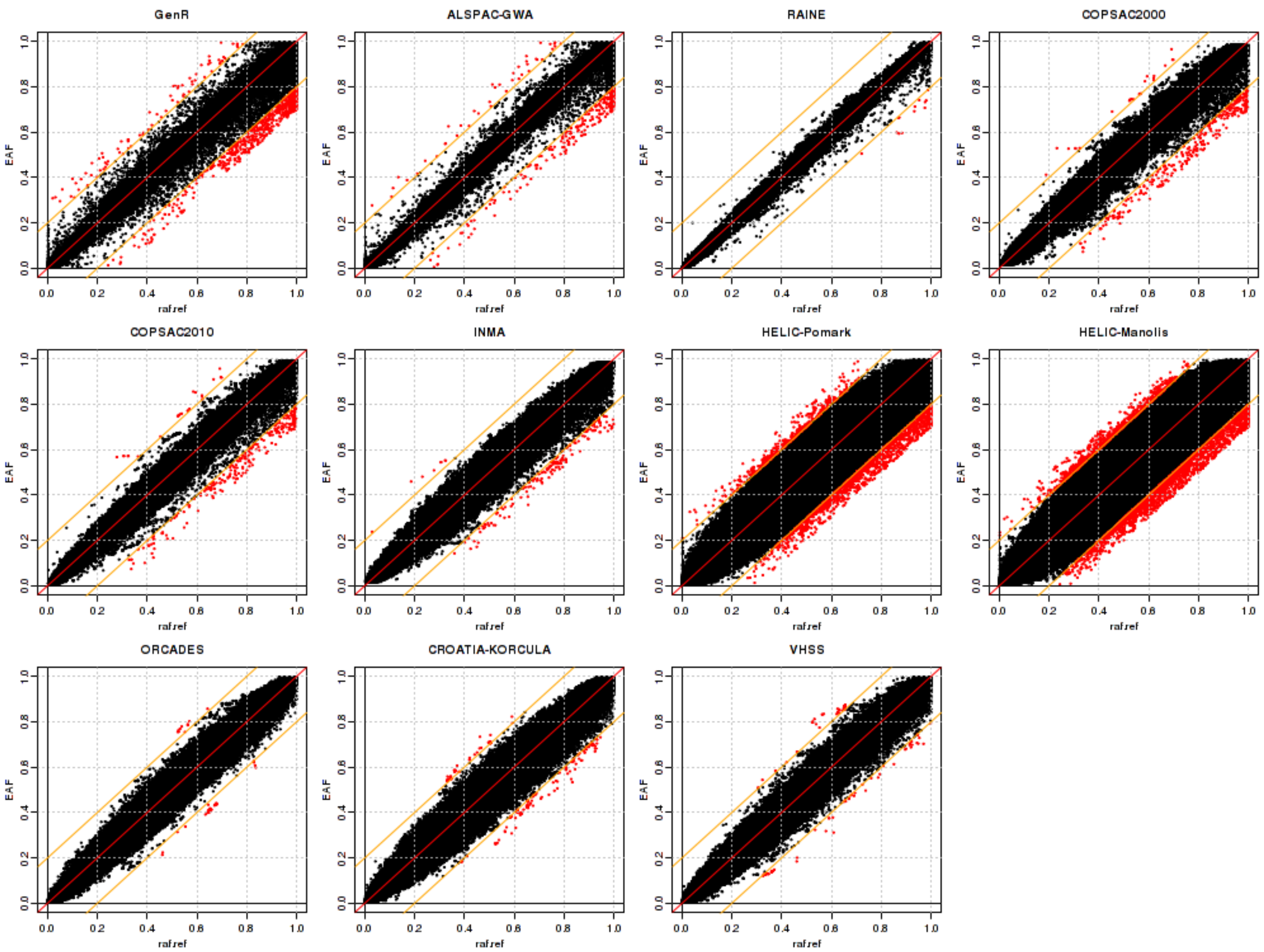

Supplementary Figure 1: Allele frequency (AF) plots for all genotyping studies in the HC analysis. In each graph, the observed effect allele frequency (EAF) in each study (y-axis) is plotted against the expected AF from the reference panel (HRC r1.1, x-axis). The red line shows the identity of observed and expected AFs. The yellow lines represent a 0.2 difference threshold between observed and expected AF, any variant that lies outside these two lines (colored in red) has a AF difference greater than 0.2.

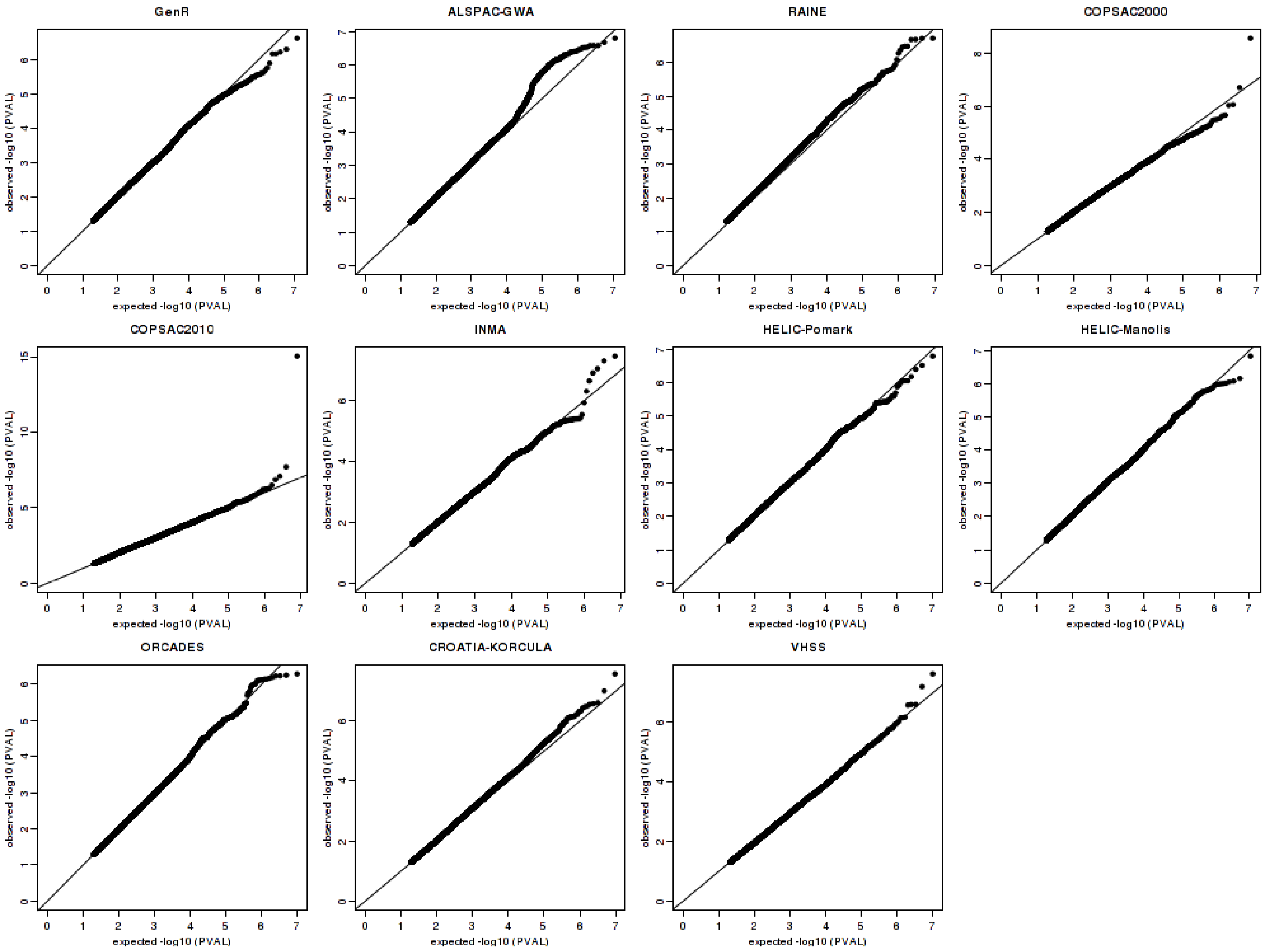

Supplementary Figure 2: **QQ plots for all genotyping studies in the HC analysis**. Each plot shows the observed p-value from the SNP association with HC (y-axis) against the expected p-value (x-axis). The black line demonstrates the null hypothesis.

Allele frequency	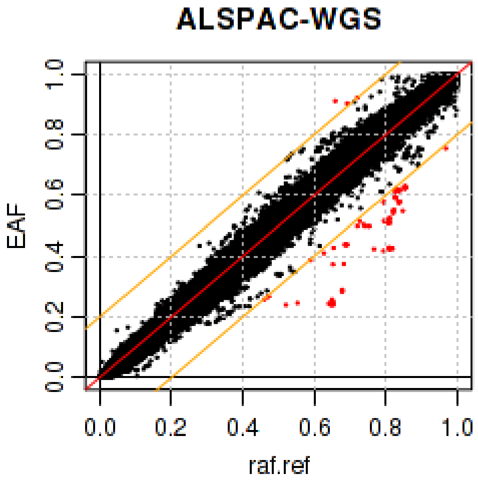
QQ plot	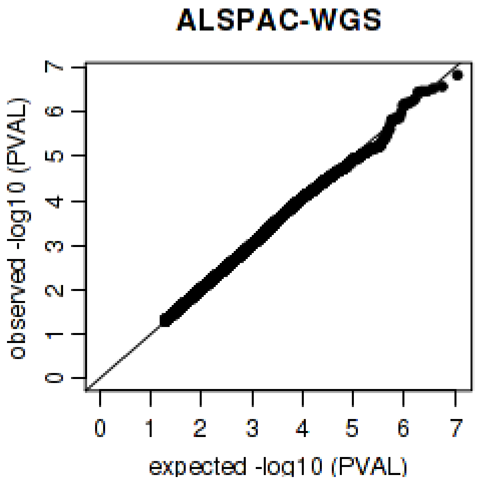

Supplementary Figure 3: Allele frequency and QQ plots for ALSPAC-WGS in the HC analysis. The observed effect allele frequency (EAF) (y-axis) is plotted against the expected AF from the reference panel (HRC r1.1, x-axis). The red line shows the identity of observed and expected AFs. The yellow lines represent a 0.2 difference threshold between observed and expected AF, any variant that lies outside these two lines (colored in red) has a AF difference greater than 0.2. The QQ plot shows the observed p-value from the SNP association with HC (y-axis) against the expected p-value (x-axis). The black line demonstrates the null hypothesis.

HC (Pediatric)	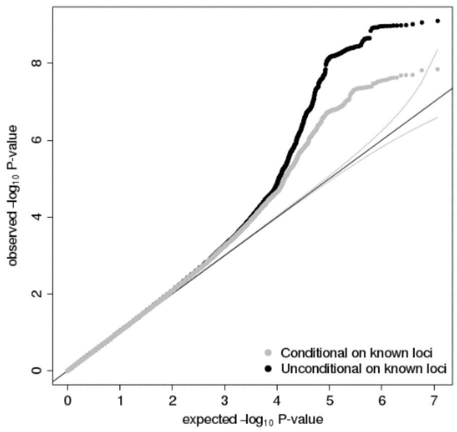
HC (Adult)	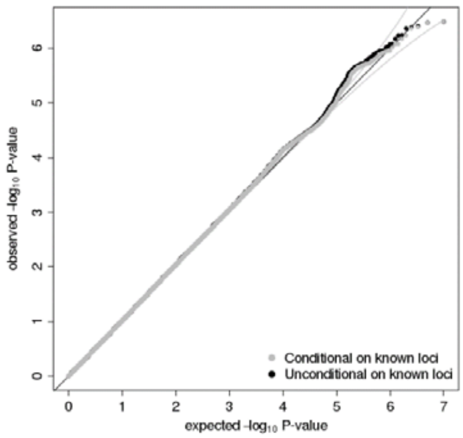
HC (Pediatric+adult)	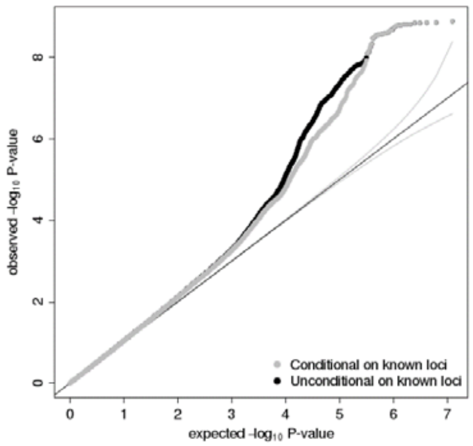

Supplementary Figure 4: QQ plots for conditional and unconditional HC meta-analyses. For each meta-analysis, the QQ plot shows the observed p-value from the SNP association with HC (y-axis) against the expected p-value (x-axis). The black line demonstrates the null hypothesis. Conditional analyses (conditioned on known loci for HC scores, brain volume and ICV) are shown in grey and unconditional analyses in black.

<table>
<tr>
<td>a

rs183336048

HC (Pediatric)</td>
<td>
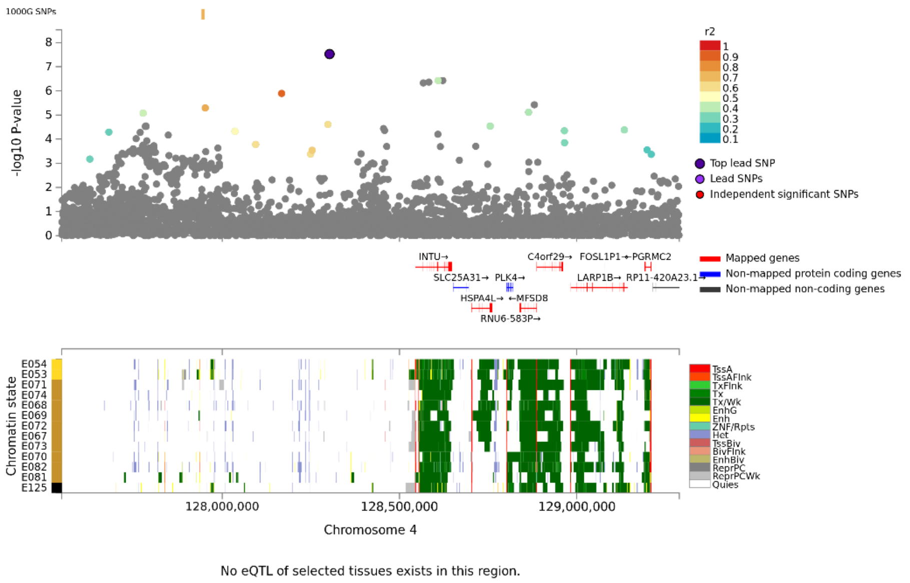

</td>
</tr>
<tr>
<td>b

rs9268812

HC
(Pediatric+adult)</td>
<td>
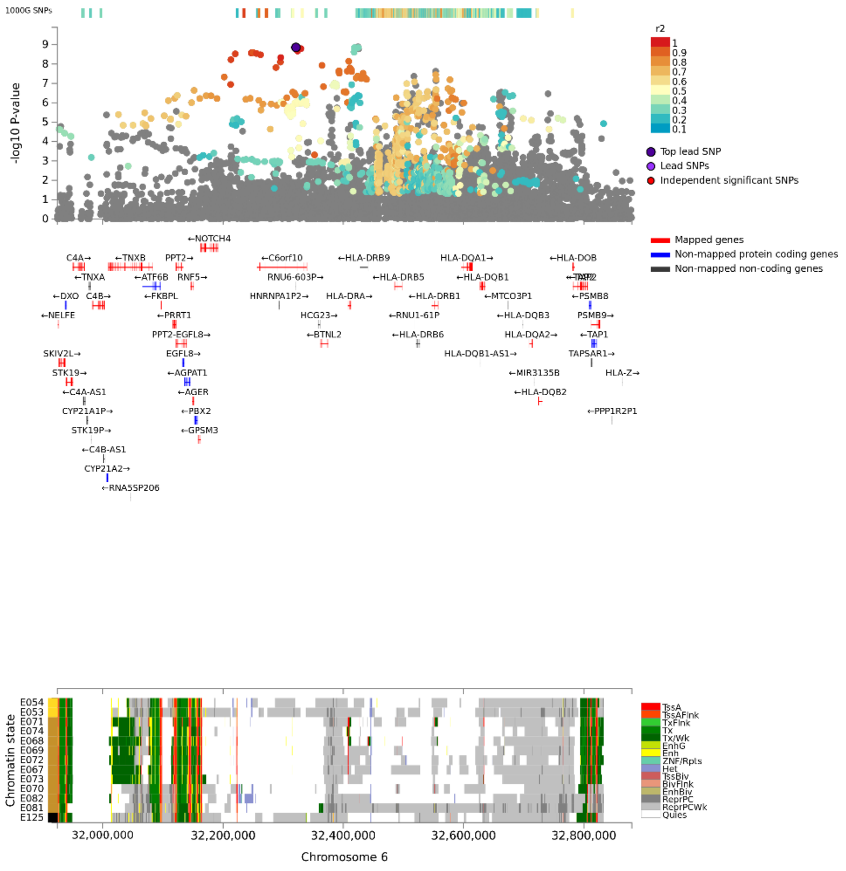

</td>
</tr>
</table>

c<br>rs35850753<br>HC<br>(Pediatric+adult)	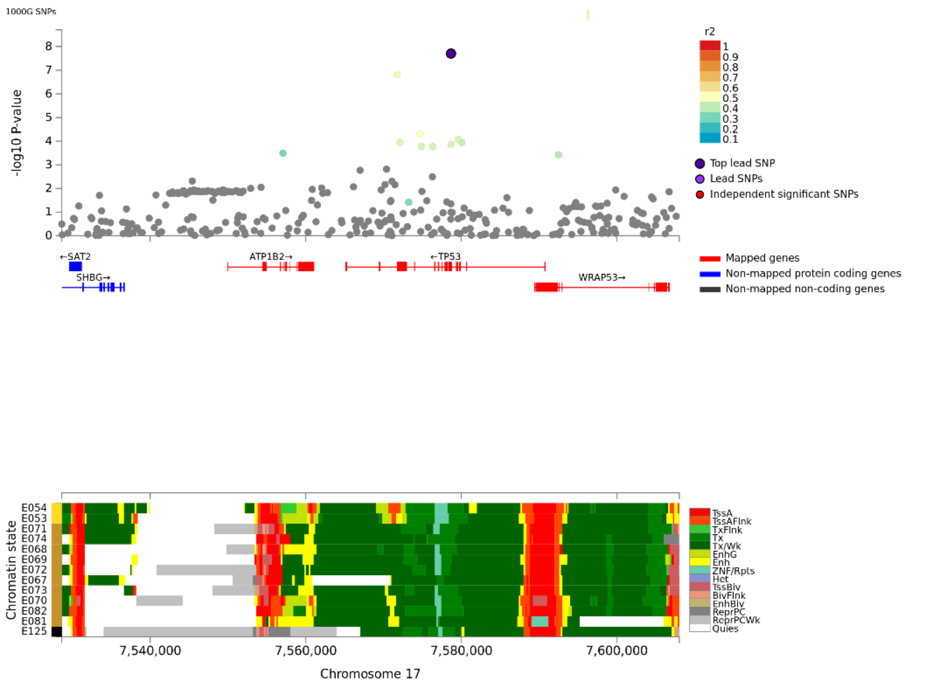

Supplementary Figure 5: Regional plots for novel HC signals. a) rs183336048, HC (Pediatric), b) rs9268812, HC (Pediatric+adult) and c) rs35850753, HC (Pediatric+adult).

Top panels: Regional association plots, all positions are reported according to NCBI Build 37 using HRC r1.1 imputed genotypes.

Lower panels: 15 Chromatin states in 13 brain tissues/cell types; Color key: *TssA* Active transcription start site (TSS), *TssAFlnk* Flanking Active TSS, *TxFlnk* Transcr. at gene 5' and 3', *Tx* Strong transcription, *Tx/Wk* Weak transcription, *EnhG* Genic enhancers, *Enh* Enhancers (Enh), *ZNF/Rpts* ZNF genes and repeats, *Het* Heterochromatin, *TssBiv* Bivalent/Poised TSS, *BivFlnk* Flanking Bivalent TSS/Enh, *EnhBiv* Bivalent Enhancer, *ReprPC* Repressed PolyComb, *ReprPCWk* Weak Repressed PolyComb, *Quies* Quiescent/Low. 15-core chromatin state from brain tissue/cell types: E054 (Neurosph) Ganglion Eminence derived primary cultured neurospheres, E053 (Neurosph) Cortex derived primary cultured neurospheres, E071 (Brain) Brain Hippocampus Middle, E074 (Brain) Brain Substantia Nigra, E068 (Brain) Brain Anterior Caudate, E069 (Brain) Brain Cingulate Gyrus, E072 Brain Inferior Temporal Lobe, E067 (Brain) Brain Angular Gyrus, E073 (Brain) Brain Dorsolateral Prefrontal Cortex, E070 (Brain) Brain Germinal Matrix, E082 (Brain) Fetal Brain Female, E081 (Brain) Fetal Brain Male, E125 (ENCODE2012) NH-A-Astrocytes Primary Cells.

All analyses were conducted using the FUMA[1] webtool.

a

rs183336048

HC (Pediatric)

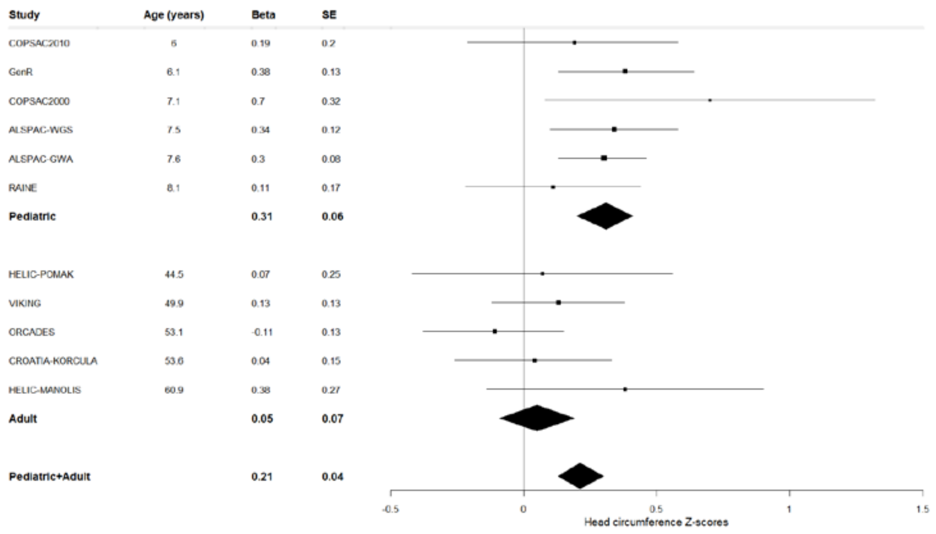

b

rs9268812

HC (Pediatric+adult)

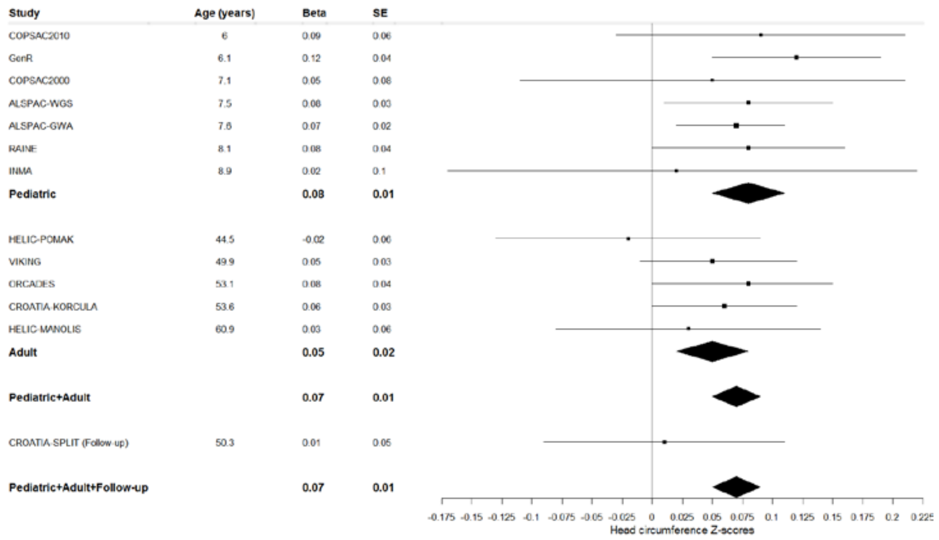

c

rs35850753

HC (Pediatric+adult)

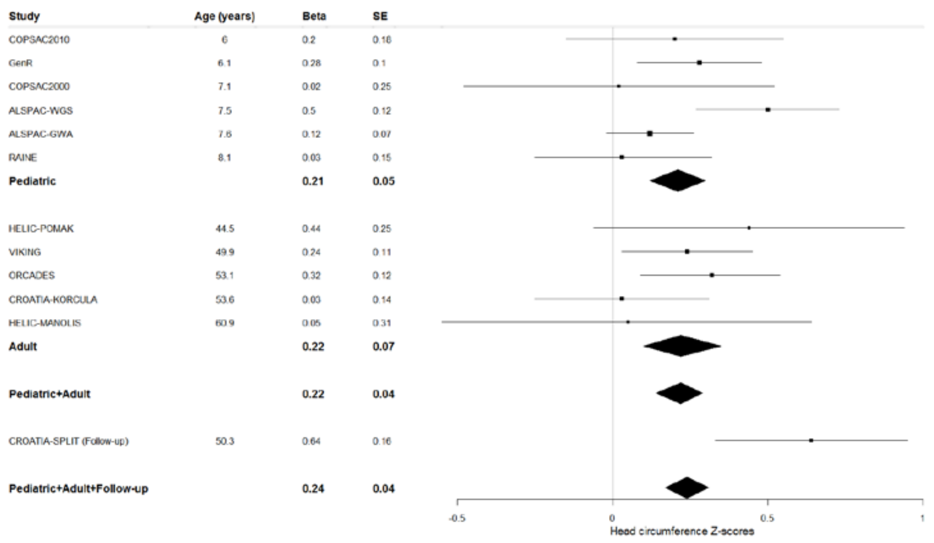

Supplementary Figure 6: Forest plots for novel HC signals. a) rs183336048, HC (Pediatric), b) rs9268812, HC (Pediatric+adult) and c) rs35850753, HC (Pediatric+adult). Age in years represents the mean age within each study; Beta is the effect size in age-adjusted standard deviation units of HC scores, normalized across males and females; SE is the standard error. Note that INMA is not included in the meta-analysis of rs183336048 and rs35850753, as these markers did not pass QC filters.

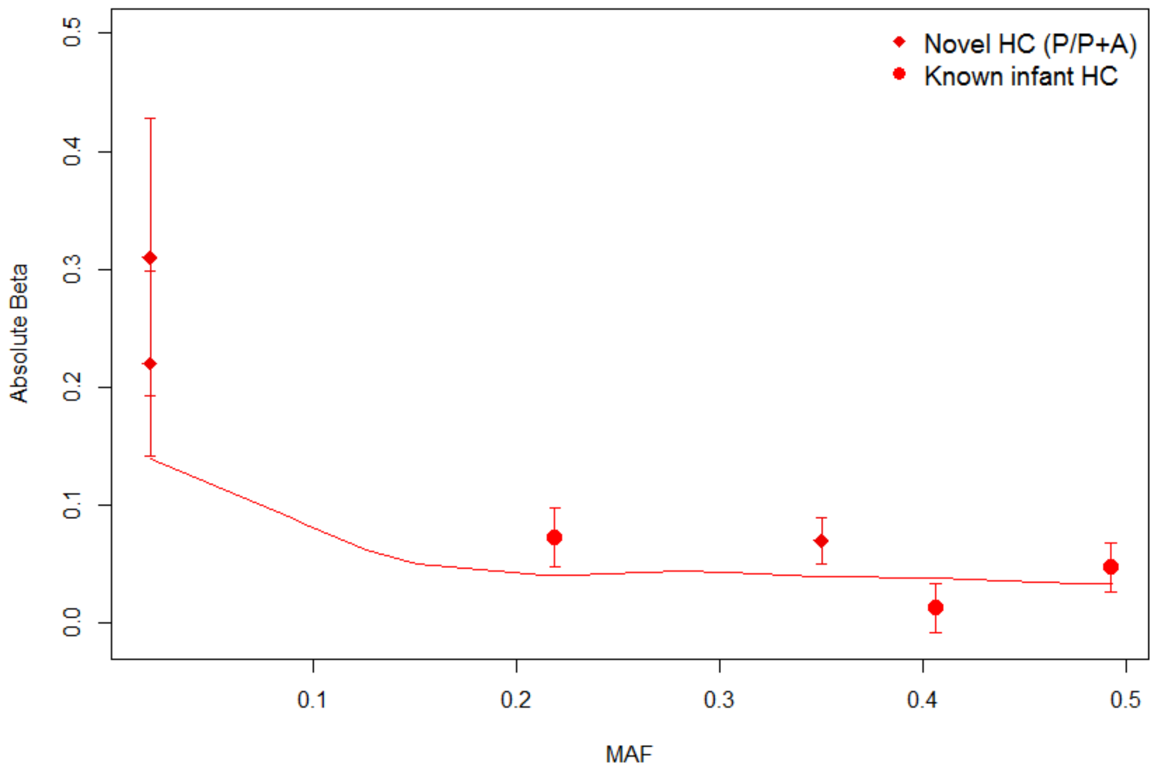

Supplementary Figure 7: Allele frequency spectrum of associated loci in the HC analysis. Effect sizes and 95% confidence intervals (absolute value of effect estimates, expressed in standard deviation units) are shown as a function of minor allele frequencies (MAF), based on the HC meta-analysis in this study. Only independent loci are included in the graph (LD: $r^2 < 0.2$ within +/- 500 kb). Novel variants are depicted as diamonds including HC (Pediatric) and HC (Pediatric + adult) analyses (denoted as P/P+A). Previously known variants on infant HC that reach nominal evidence for genome-wide significance ($p<5x10^{-8}$, two-sided) are denoted as circles (Supplementary Data 4).

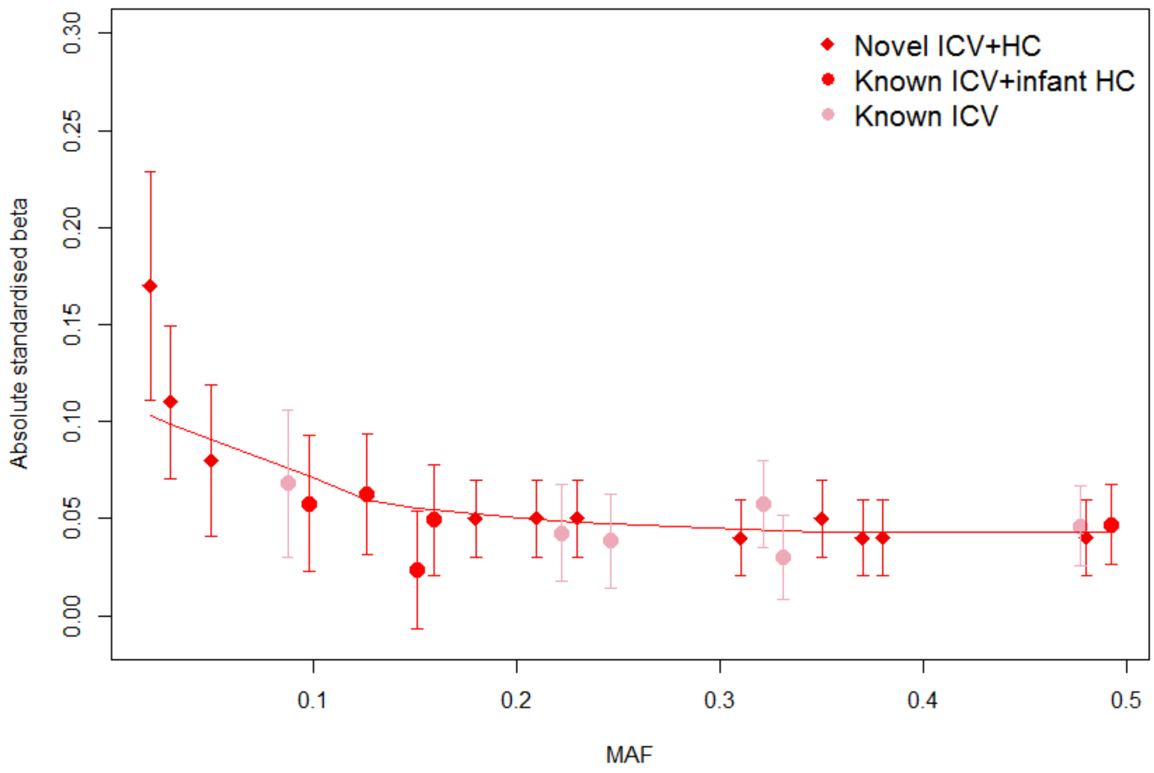

Supplementary Figure 8: Allele frequency spectrum of associated loci in the combined ICV+HC analysis. Effect sizes and 95% confidence intervals (absolute value of standardized effect estimates, expressed in standard deviation units) are shown as a function of minor allele frequencies (MAF), based on the combined ICV and HC meta-analysis in this study. Only independent loci are included in the graph (LD: $r^2 < 0.2$ within +/- 500 kb). Novel variants identified in a combined ICV+HC analysis in this study are depicted as diamonds, and previously known variants that reach nominal evidence for genome-wide significance ($p<5x10^{-8}$, two-sided) are denoted as circles. Latter includes previously reported findings from a combined analysis for ICV+infant HC as well as loci identified both for ICV and infant HC (Supplementary Table 4)

Supplementary Tables

Supplementary Table 1: HC cohort information for genome-wide analysis discovery samples

	Study (short name)	Study (full name)	Country of origin[a]	Genotyping platform(s)	Genotype source	Association software	N	Age Mean(SD) [range]	% Male	HC (cm) Mean (SD) [range]	GC (λ)
Pediatric	ALSPAC-WGS	The Avon Longitudinal Study of Parents and Children	UK	-	WGS	SNPtest v2.5	1,762	7.5 (0.1) [7.1;8.8]	49.4	52.6(1.43) [48;58]	1.007
Pediatric	ALSPAC-GWA	The Avon Longitudinal Study of Parents and Children	UK	Illumina Human Hap550	UK10K/ 1KGP	SNPtest v2.5	4,388	7.6 (0.35) [6.9;9.5]	52.8	52.5(1.45) [48;58]	1.023
Pediatric	GenR	Generation R Study	NL	Illumina 610 Quad and 660	UK10K/ 1KGP	SNPtest v2.5	1,995	6.1 (0.4) [4.8;9.0]	50.7	51.5 (1.50) [47;57]	1.01
Pediatric	Raine	Western Australia Pregnancy Cohort (Raine) Study	Australia	Illumina 660W Quad BeadChip	HRC r1.1	ProABEL v. 0.4.4	1303	8.11 (0.35) [6.6;10.0]	51.2	53.14 (1.54) [48.0-58.6]	1.074
Pediatric	COPSAC2000	Copenhagen Prospective Study on Asthma in Children	Denmark	Illumina Omni-Express Exome	HRC r1.1	SNPtest v2.5	296	7.12 (0.35) [6.9:9.3]	49.7	52.6(1.51) [48;58]]	1.023
Pediatric	COPSAC2010	Copenhagen Prospective Study on Asthma in Children	Denmark	Illumina Omni-Express Exome	HRC r1.1	SNPtest v2.5	559	6.00 (0.17) [5.5;7.0]	51.2	52.11(1.44)[48.2;57 .9]	1.015
Pediatric	INMA	Infancia y Medio Ambiente	Spain	Illumina Human Omni1 Quad Beadchip	HRC r1.1	SNPtest v2.5	297	8.94 (0.62) [7.29;10.71]	53.4	52.31(1.51) [47.5;57.0]	1.003
	**Pediatric HC**						10600				
Adult	HELIC-Pomak	Hellenic Isolated Cohorts - Pomark	Greece	IlluminaOmniExpress 770KBeadChip, Illumina Exome Chip	UK10K/ 1KGP	GEMMA	847	44.5 (13.7) [16;87]	30.3	55.5 (1.96) [48;63]	1.01
Adult	HELIC-MANOLIS	Hellenic Isolated Cohorts - MANOLIS	Greece	lluminaOmniExpress 770KBeadChip and Illumina Exome Chip	UK10K/ 1KGP	GEMMA	1,055	60.9 (19.6) [18;98]	43.2	55.4 (2.48) [47;66]	1.007
Adult	ORCADES	Orkney Complex Disease Study	UK	Illumina Hap300v2 or 370CNV - Quad beadchips, Illumina Omni1, Illumina OmniExpress beadchips	HRC r1.1	REGSCAN v0.2	1,689	53.06 (14.82) [16.5-88.5]	39.5	56.2 (1.98) [50.6-62.4]	0.998
Adult	CROATIA-KORCULA	10001 Dalmations: The Croatian Biobank	Croatia	Illumina CNV370v1, CNV370-Quadv3	HRC r1.1	GenABEL REGSCAN v0.2	2,601	53.60 (15.57) [18;98]	36.3	56.6 (2.01) [48.5;64.0]	1.01
Adult	VIKING	The Viking Health Study - Shetland	UK	Illumina OmniExpressExome8v1-2_A	HRC r1.1	REGSCAN v0.2	2,092	49.87 (15.2) [18.1;91.8]	39.9	56.46 (1.96) [49.9;63.4]	0.988
	**Adult HC**						8281				
	**Pediatric + adult HC**						18881				

a - all samples are of European ancestry; Age in years; GC - Genomic control factor; HC - Head circumference; HRC - The Haplotype Reference Consortium imputation template; UK10K/1KG - UK10K+1KGP combined imputation reference template; WGS - Whole Genome Sequencing

Ethical approval (discovery cohorts)

- ALSPAC-WGS, ALSPAC-GWA: ALSPAC Law-and-Ethics Committee and the Local Research-Ethics Committees
- GenR: Medical Ethics Committee of the Erasmus MC, University Medical Center, Rotterdam
- Raine: Human Research Ethics Committees at the University of Western Australia and Curtin University
- COPSAC2000, COPSAC2010: Copenhagen Ethics Committee and the Danish Data Protection Agency
- INMA: Clinical Research Ethical Committee of the Medical Assistance Municipal Institute Barcelona
- HELIC-Pomak and HELIC-MANOLIS: Harokopio University Bioethics Committee
- ORCADES: Research Ethics Committees in Orkney and Aberdeen (North of Scotland Research Ethics Committee)
- CROATIA-KORCULA: Ethics committees of the Medical School of the University of Zagreb, the Medical School of the University of Split and the National Health Service Lothian
- VIKING: South East Scotland Research Ethics Committee

Supplementary Table 2: HC cohort information for the follow-up sample (Adult)

Study (short name)	Study (full name)	Country of origin[a]	Genotyping platform(s)	Genotype source	Association software	N	Age Mean(SD) [range]	% Male	HC (cm) Mean (SD) [range]	Genomic control factor (λ)
CROATIA-SPLIT	The Croatian Biobank, Split	Croatia	370CNV-Quadv3, Illumina OmniExpress Exome-8v1_A beadchips	1KGP phase 1 V3	ProABEL	973	50.34 (14.39) [18-85]	39.2	56.2 (2.04) [50.0-63.2]	1.007

a - all samples are of European ancestry; Age in years; HC - Head circumference; 1KGP - 1000 genomes

Ethical approval (replication cohort)

CROATIA-SPLIT: Ethics committees of the Medical School of the University of Zagreb, the Medical School of the University of Split and the National Health Service Lothian

Supplementary Table 3: Association between rs35850753 and HC growth

SITAR random effect	Allele	β	SE	p	Interpretation of SITAR random effect
a	rs35850753_T	0.29	0.04	6.9E-12	random intercept relative to the spline curve intercept (mean)
c	rs35850753_T	0.01	0.0015	7.1E-11	HC velocity

Linear regression of SITAR random effects for differences in mean head circumference and growth velocity on allele dosage (rs35850753). P-values are two-sided (Wald-test). HC - Head circumference; SITAR - Super Imposition by Translation And Rotation model

Supplementary Table 4: Known signals for HC and ICV

Variant	Chr	Position(b37)	Trait [reference]	Reported *p* for genetic association
rs6734848	2	183866063	ICV and infant HC[2]	6.0E-09
rs2271386	3	141712708	ICV and infant HC[2]	4.5E-09
rs9811910	3	190670902	ICV[2]	2.0E−09
rs2022464	6	108945370	ICV[2]	3.7E−11
rs11759026	6	126792095	ICV[2]	2.2E−20
rs4273712	6	126964510	ICV[3]	1.8E−13
rs7802256	7	33111950	ICV and infant HC[2]	4.6E-08
rs3779381	7	120966790	Brain volume[4]	2.0E-16
rs2536185	7	120984041	Brain volume[4]	1.3E-16
rs11191683	10	105170649	ICV[2]	1.1E−10
rs10784502[a]	12	66343810	ICV[5]	1.1E−12
rs1042725	12	66358347	Infant HC[6]	2.8 E-10
rs138074335[b]	12	66374247	ICV[2]	6.2E−09
rs2195243	12	102922986	ICV[2]	1.5E−08
rs7980687	12	123822711	Infant HC[7]	8.1E−9
rs11655470	17	43795433	Infant HC[7]	1.4E−6
rs17689882[c]	17	43906828	ICV	7.7E−9
rs9303525	17	44187257	ICV[3]	7.6E−15
rs199525	17	44847834	ICV[2]	3.8E−21
rs10483213	22	42339525	ICV and infant HC[2]	4.0E-08

a - Omitted from conditional analysis due to high LD with rs1042725
b - Low reported imputation quality (INFO score > 0.6) in most studies
c - Omitted from conditional analysis due to correlation with other variants
HC - Head circumference; ICV - Intracranial volume

Supplementary Table 5: Predicted gene expression for HC

Meta-analysis	Tissue	Gene	Gene	Z	β	*p*	var_g	pred_perf_R2	pred_perf_P	pred_perf_q	n_snps_in_model
HC (Pediatric)	Whole blood	ENSG00000204469.8	*PRRC2A*	-5.00	-0.27	5.73E-07	0.03	0.09	1.51E-08	1.70E-08	5
HC (Pediatric+adult)	Whole blood	ENSG00000204469.8	*PRRC2A*	-4.88	-0.21	1.08E-06	0.03	0.09	1.51E-08	1.70E-08	5
HC (Pediatric+adult)	Whole blood	ENSG00000206344.6	*HCG27*	-4.80	-0.15	1.62E-06	0.06	0.18	4.17E-16	9.38E-16	36
HC (Pediatric+adult)	Cerebellum	ENSG00000206344.6	*HCG27*	-4.87	-0.16	1.12E-06	0.06	0.15	4.15E-05	9.33E-05	61

S-PrediXcan analysis for enrichment in whole blood, cerebellum and cortex, as implemented in MetaXcan standalone software (v0.3.5). Only signals passing a multiple testing threshold of p<2.25E-06) are shown. Full data are provided in Supplementary Data 8.

Supplementary Table 6: Unconstrained LD-score regression analysis

Sample	HC (Pediatric)	HC (Adult)	HC (Pediatric+adult)
LDSC h^2 (SE)	0.31 (0.05)	0.097 (0.06)	0.21 (0.03)
Intercept	1.00 (0.01)	1.00 (0.01)	1.00 (0.01)
Lambda GC	1.0557	1.0165	1.0679
SNPs	1137401	1178563	1174943

LD score regression was performed with LDSC software (v1.0.0).

Supplementary Table 7: Genetic-relationship-matrix structural equation modelling of HC

Path	β	SE	Z	p
a11	0.59	0.06	10.44	1.6E-25
a21	0.59	0.06	9.91	3.7E-23
a31	0.49	0.07	6.77	1.3E-11
a22	0.30	0.09	3.43	6.0E-04
a32	0.23	0.12	1.97	4.9E-02
a33	-0.30	0.05	-5.71	1.1E-08
e11	0.81	0.04	19.43	4.4E-84
e21	0.48	0.05	10.09	6.0E-24
e31	0.44	0.06	7.73	1.1E-14
e22	-0.58	0.03	-17.82	5.3E-71
e32	-0.44	0.05	-8.97	2.8E-19
e33	-0.47	0.03	-13.97	2.4E-44

Genetic-relationship-matrix structural equation modelling using a Cholesky decomposition. Path coefficients of the full Cholesky decomposition model using longitudinal head circumference (HC) measures from ALSPAC (1.5 years, N =3,945; 7 years, N=5,819; 15 years, N=3,406) are shown. Phenotypic variance (P1, P2, P3) was dissected into three genetic (A1, A2 and A3) and three residual (E1, E2 and E3) factors. All estimates were standardized.

Supplementary Table 8: Unconstrained LD-score genetic correlation analysis

Trait	PMID	HC (pediatric)				HC (Adult)				HC (pediatric+Adult)			
		r_g	SE	Z	*p*	r_g	SE	Z	*p*	r_g	SE	Z	*p*
Birth length	25281659	0.48	0.10	4.58	4.6E-06	0.26	0.22	1.18	0.24	0.42	0.09	4.64	3.4E-06
Birth weight (2013)	23202124	0.51	0.12	4.30	1.7E-05	0.51	0.25	1.99	0.05	0.50	0.11	4.47	8.0E-06
Birth weight (2016)	27680694	0.43	0.06	6.87	6.5E-12	0.44	0.17	2.55	0.01	0.44	0.06	7.81	5.5E-15
Infant HC	22504419	1.00	0.13	10.60	2.8E-26	0.59	0.31	1.91	0.06	1.16	0.12	9.51	1.8E-21
Late childhood height	23449627	0.42	0.09	4.78	1.8E-06	0.72	0.27	2.67	7.6E-03	0.51	0.09	5.83	5.6E-09
Adult height (2010)	20881960	0.30	0.05	5.97	2.4E-09	0.44	0.15	2.91	3.7E-03	0.34	0.05	7.09	1.4E-12
Extreme height	23563607	0.28	0.07	4.26	2.0E-05	0.61	0.41	1.47	0.14	0.34	0.07	4.62	3.9E-06
Body mass index	20935630	0.25	0.05	4.75	2.0E-06	0.28	0.12	2.28	0.02	0.25	0.05	5.54	3.1E-08
Overweight	23563607	0.23	0.06	3.92	8.7E-05	0.35	0.15	2.37	0.02	0.27	0.06	4.54	5.5E-06
Childhood obesity	22484627	0.41	0.09	4.59	4.4E-06	0.34	0.18	1.83	0.07	0.37	0.08	4.61	4.0E-06
Obesity (Class1)	23563607	0.22	0.06	3.83	1.0E-04	0.35	0.15	2.35	0.02	0.25	0.06	4.38	1.2E-05
Hip circumference	25673412	0.44	0.06	7.41	1.2E-13	0.30	0.13	2.24	0.03	0.39	0.06	6.93	4.1E-12
Waist circumference	25673412	0.35	0.05	6.36	2.0E-10	0.28	0.13	2.13	0.03	0.33	0.05	6.08	1.2E-09
Years of schooling (2014)	25201988	0.28	0.07	3.87	1.0E-04	0.07	0.13	0.53	0.60	0.22	0.06	3.35	8.0E-04
Years of schooling (2016)	27225129	0.22	0.04	5.27	1.3E-07	0.08	0.09	0.88	0.38	0.17	0.04	4.18	2.9E-05
Intelligence	28530673	0.36	0.06	6.26	3.8E-10	0.26	0.14	1.82	0.07	0.32	0.06	5.67	1.4E-08
ICV	25607358	0.77	0.16	4.92	8.8E-07	1.00	0.49	2.49	0.01	0.91	0.16	5.65	1.6E-08

Genetic correlation (r_g) estimates for 14 out of 235 traits passing a multiple testing threshold of 1.4E-04 in at least one MA are shown, as provided by LDHUB v1.9.0; estimates not passing the threshold are shown in grey; r_g values > 1 were truncated to one. Full data for HC (Pediatric+adult) are provided in Supplementary Data 9.
HC - Head circumference

Supplementary Notes

1. Cohort descriptions

Discovery stage

The Avon Longitudinal Study of Parents and Children (ALSPAC) is a longitudinal pregnancy-ascertained UK population-based birth cohort. The study includes more than 14,000 mothers and their children (estimated birth date: 1991 to 1992)[8,9]. During the course of this longitudinal study, ALSPAC families have provided a large amount of genetic and environmental information on mothers, fathers and children. The study website contains details of all collected data through a fully searchable data dictionary (http://www.bris.ac.uk/alspac/researchers/data-access/data dictionary/). Ethical approval was obtained from the ALSPAC Law-and-Ethics Committee (IRB00003312) and the Local Research-Ethics Committees, written informed consent was obtained from a parent or individual with parental responsibility and assent (and for older children consent) was obtained from the child participants. ALSPAC children and adolescents for this study were selected to maximize phenotypic coverage as well as information on previous genome-wide chip genotyping data, other “-omic” datasets (transcriptomic, metabolomic) and consent to whole genome sequencing. Head circumference (HC) was measured to the nearest mm at the widest horizontal circumference of the head using the Harpenden anthropometric tape at the age of seven years (Supplementary Table 1).

DNA samples from 2,040 ALSPAC children were submitted for whole-genome sequence data (**WGS**), of which 1,976 samples were sequenced and subjected to a stringent quality control and variant calling procedure. Samples were removed from the analysis because of any of the following reasons, as previously described[10]: high overall discordance between WGS and GWAS genotype data, high heterozygosity, missing GWAS genotype data or a low mean read-depth (below 4x). 1,928 ALSPAC samples were brought forward to the genotype refinement step. Post-refinement quality control included further exclusions due to non-reference discordance with GWAS data, cryptic relatedness or sex-discordance. The final data set included 1,867 ALSPAC children of which 1,762 had both information on WGS and HC scores at age 7 (Supplementary Table 1). All variants were restricted to a MAF of > 0.25% or an allele count (AC) >= 4. Variants from cohorts with N<500 were restricted to have MAF> 0.05.

Additional genome-wide analysis (**GWA**) data was available for 6,631 unrelated ALSPAC children from the Illumina HumanHap550 quad chip genotyping platform. After pre-imputation quality control (QC; sex-discordance, heterozygosity, non-European ancestry, cryptic relatedness), 6,557 children were retained of which 4,388 also had information on HC (Supplementary Table 1). Genotype imputation was carried out into a UK10K haplotype reference panel as previously described[11].

ALSPAC genotype and phenotype data can be requested through the ALSPAC Executive Committee following a procedure described at http://www.bristol.ac.uk/alspac/researchers/access/.

The **HELIC** (Hellenic Isolated Cohorts; www.helic.org) cohorts comprise two adult Greek populations-based cohorts, **HELIC MANOLIS** (Minoan Isolates) and **HELIC Pomak**. HELIC MANOLIS represents a collection of adult inhabitants from Anogia and surrounding Mylopotamos villages), while HELIC Pomak was sampled from the Pomak villages, a set of isolated mountainous villages in the North of Greece. Both HELIC cohorts collected biological samples for DNA extraction and information from lab-based blood measurements and interview-based questionnaires. This includes anthropometric and biometric measurements, clinical evaluation data, biochemical and haematological profiles, self-reported medical history, demographic, socioeconomic and lifestyle information. The study was approved by the Harokopio University Bioethics Committee and informed consent was obtained from every participant. HC scores were measured to the nearest 0.1 cm at the widest horizontal circumference of the head using a measuring tape. The tape was applied above ears, across the glabella region, and at the top of the occipital region backwards.

The HELIC samples were genotyped using both the Illumina HumanOmniExpress and Illumina HumanExome chip at the Wellcome Trust Sanger Institute as previously described. QC steps included, in brief, the exclusion of samples with sex discrepancies or low call rate, duplicates, ethnic outliers and heterozygosity outliers. In addition, variants with call rate <95%, a MAF<5%, MAF differences compared to variants of the 1000 Genomes Project, deviations from Hardy-Weinberg equilibrium ($p<10^{-4}$) were filtered out as well monomorphic, duplicate or singleton markers. Genotypes for the OmniExpress and Exome chip were combined into a single dataset. Genotype data was phased using SHAPEITv2 and imputed using IMPUTEv2 against a UK10K haplotype reference panel.1,055 participants from HELIC MANOLIS and 847 participants form HELIC Pomak had both genotype and HC information available (Supplementary Table 1).

Genotype data from the HELIC cohorts have been deposited in the European Genome-phenome Archive (https://www.ebi.ac.uk/ega/home): EGAD00010000518; EGAD00010000522; EGAD00010000610; EGAD00001001636. Phenotypic data are available on request from the Principal Investigators, Eleftheria Zeggini (Eleftheria@sanger.ac.uk) and Georgios Dedoussis (dedousi@hua.gr).

GenerationR (GenR) is a population-based prospective cohort study from fetal life onwards in Rotterdam, the Netherlands. The Medical Ethics Committee of the Erasmus MC, University Medical Center, Rotterdam, has approved the study and written informed consent was obtained for all participants. Participating children were born between April 2002 and January 2006. Enrollment was aimed at early pregnancy, but was allowed until birth of the child. In total, 9,778 mothers and their children were included in the study and details on recruitment and data collection have been described in detail elsewhere. Cord blood for DNA isolation was available in 58% of all live-born participating children. Missing cord blood samples were mainly due to logistical constraints at the delivery. Sex-mismatch rate between genome based sex and midwife-record based sex was low (<0.5%), indicating that possible contamination of maternal DNA was extremely low. HC was measured once with a measuring tape (SECA, Hamburg, Germany), above the ears and eyebrows, at the largest diameter.

GenR participants were genotyped using the Illumina 610 Quad and 660 genotyping platforms. Prior to imputation, variants were excluded because of high levels of missingness (SNP call rate <98%), strong departures from Hardy-Weinberg equilibrium ($p < 10^{-6}$), or low MAF (<0.1%). In addition, samples were excluded because of sex discordance, high heterozygosity, low call rate (<97.5%) or because they were duplicates. Genotype data was imputed using IMPUTEv2 against a UK10K haplotype reference panel.

The Generation R Study has an open policy in regard to collaboration with other research groups. Requests for collaboration should primarily be pointed to Prof. dr. Vincent Jaddoe (v.jaddoe@erasmusmc.nl). These requests are discussed in the Generation R Study Management Team regarding their study aims, overlap with ongoing studies, logistic consequences and financial contributions.

The **Western Australian Pregnancy Cohort (Raine) Study** is a prospective pregnancy birth cohort based. Women living in Western Australia were recruited in pregnancy (before 18 weeks gestation) between 1989 and 1991. 2,900 women were recruited, with 2,868 live births from 2,826 mothers. Follow up has been conducted at regular intervals up to 23 years of age and is ongoing. The study design and methods for the 22 year follow up are described in Straker et al.[12]. As described, approval for the 22 year follow-up was obtained from the Human Research Ethics Committees at the University of Western Australia (RA/4/1/5202) and Curtin University (HR67/2013). The study was carried out in accordance with the Declaration of Helsinki and informed consent was obtained from all participants[12].

Raine participants were genotyped using the Illumina 660W Quad BeadChip. Prior to imputation variants were excluded because of missingness (SNP call rate <95%), strong departures from Hardy-

Weinberg equilibrium ($P < 5.7 \times 10^{-7}$), or low MAF (<1%). Participants were excluded prior to imputation if they had poor overall call rates (<97%), for gender discrepancy or high degree of cryptic relatedness (pi > 0.1875).

The Raine Study encourages collaboration with national and international researchers. More details about the Raine Study, data, and how to get access to the data are published on the website of the Raine Study (www.rainestudy.org.au).

The **Infancia y Medio Ambiente (INMA)** is a collection of prospective cohort studies which recruited pregnant women from seven study areas in Spain with the aim of studying the relationship between environmental exposure and pollutants on children's development and health. Individual studies recruited between 1997 and 2008, giving a total of 3,916 pregnant women in the INMA collection and 3,887 births. The study was approved by the Clinical Research Ethical Committee of the Medical Assistance Municipal Institute Barcelona. Study participants gave valid consent. The INMA project is described in Guxens et al.[13]. For the current study only participants from INMA Sabadell were considered.

INMA participants were genotyped using the Illumina HumanOmni1 Quad Beadchip assay. Prior to imputation variants were excluded because of missingness (call rate <95%), deviation from Hardy-Weinberg equilibrium ($p < 10^{-6}$) or low MAF (<1%). Individuals were removed for poor overall call rates (<98%). In addition, the younger sibling of any sibling pair in the study was removed prior to imputation.

INMA data access is through a system of managed open access. The steps to apply for access to the data included in this research and all other INMA data are described in full at http://www.proyectoinma.org/presentacion-inma/politica-colaboracion/en_politica-colaboracion.html.

The **Copenhagen Prospective Studies on Asthma in Childhood (COPSAC)** is a pair of single center prospective birth cohort studies in Copenhagen area, Denmark where $COPSAC_{2000}$ study comprises of children born to asthmatic mothers. Two cohorts are currently active - $COPSAC_{2000}$ (which recruited pregnant women with due dates between 1998 and 2001) and $COPSAC_{2010}$, (which recruited between 2009 and 2010). The study design for $COPSAC_{2000}$ and $COPSAC_{2010}$ have been described previously[14,15]. The studies are conducted in accordance with the Declaration of Helsinki and were approved by the Copenhagen Ethics Committee (KF01-289/96) and the Danish Data Protection Agency (1996-1200-360), and participants provided informed consent[14,15].

The COPSAC participants were genotyped using the Illumina Omni-Express Exome. Prior to imputation variants were excluded because of missingness (call rate <98%), deviation from Hardy-Weinberg equilibrium ($p < 10^{-6}$) or low MAF (<1%). Individuals were removed for poor overall call rates (<95%). In addition, ethnic outliers, duplicates, gender mismatch and related individuals in the study were removed prior to imputation.

COPSAC cohorts (COPSAC2000 and COPSAC2010) data access is through request. To access the data included in this research a request to Tarunveer S. Ahluwalia (tarun.ahluwalia@dbac.dk) or Klaus Bønnelykke (kb@copsac.com) can be made. All other COPSAC data are described in brief at http://copsac.com/available-data/.

The **Orkney Complex Disease Study (ORCADES)** is a family-based, cross-sectional study that seeks to identify genetic factors influencing cardiovascular and other disease risk in the isolated archipelago of the Orkney Isles in northern Scotland[16]. Genetic diversity in this population is decreased compared to Mainland Scotland, consistent with the high levels of endogamy historically. 2078 participants aged 16-100 years were recruited between 2005 and 2011, most having three or four

grandparents from Orkney, the remainder with two Orcadian grandparents. Fasting blood samples were collected and many health-related phenotypes and environmental exposures were measured in each individual. All participants gave written informed consent and the study was approved by Research Ethics Committees in Orkney and Aberdeen (North of Scotland REC).

ORCADES participants' DNA was genotyped using the Illumina HumanHap300v2 or 370CNV-Quad beadchips (n=890), the Illumina Omni1 (n=304) or Illumina OmniExpress beadchips (n=1073). Individuals were removed for poor overall call rates (<97%). In addition, excess heterozygosity, gender discrepancies and ethnic outliers in the study were checked. Prior to imputation, variants were excluded because of missingness (call rate <98%), deviation from Hardy-Weinberg equilibrium ($p < 10^{-6}$) or low MAF (<1%) and curated using the HRC preparation checking tool available from (https://imputation.sanger.ac.uk/). Imputation was carried out separately for the low-density and the higher density chips and genotype data merged post-imputation. Data was first phased using SHAPEITv2.r873 with the duoHMM function and imputed on the SANGER imputation server (https://imputation.sanger.ac.uk/) using the PBWT software against the haplotype HRC.r1.1 reference panel. Post imputation, monomorphic, duplicated and low imputation quality variants (INFO < 0.4) were removed.

The **Viking Health Study - Shetland (VIKING)** is a family-based, cross-sectional study that seeks to identify genetic factors influencing cardiovascular and other disease risk in the population isolate of the Shetland Isles in northern Scotland. Genetic diversity in this population is decreased compared to Mainland Scotland, consistent with the high levels of endogamy historically. 2105 participants were recruited between 2013 and 2015, each having at least three grandparents from Shetland. Fasting blood samples were collected and many health-related phenotypes and environmental exposures were measured in each individual. All participants gave written informed consent and the study was approved by the South East Scotland Research Ethics Committee. The Shetland VHSS participants' DNA was genotyped using the Illumina HumanOmniExpressExome8v1-2_A and genotyping quality controls and imputation carried out as described for the ORCADES.

CROATIA-KORCULA is a population-based, cross-sectional study carried out on the Dalmatian island of Korčula and part of a larger cohort, the 100001 Dalmatians: The Croatian Biobank[17]. The study received approval from the ethics committees of the Medical School of the University of Zagreb, the Medical School of the University of Split and the National Health Service (NHS) Lothian, and followed the tenets of the Declaration of Helsinki. The study includes 2833 participants in total from three recruitment phases in 2007, 2013 and 2014. Genotyping was performed using the Illumina CNV370v1 and CNV370-Quadv3 (Phase1 N=969) or OmniExpressExome-8v1-2_A (Phase2 and 3 N=1815). Genotyping quality controls and imputation were carried out as described for the ORCADES.

Follow up phase

CROATIA-SPLIT is a population-based, cross-sectional study which is also part of the 100001 Dalmatians: The Croatian Biobank[17], with 1012 individuals, aged 18-95, recruited in the city of Split. The study received approval from the ethics committees of the Medical School of the University of Zagreb, the Medical School of the University of Split and the National Health Service (NHS) Lothian, and followed the tenets of the Declaration of Helsinki. The CROATIA-SPLIT participants were genotyped roughly half and half using the 370CNV-Quadv3 and Illumina OmniExpress Exome-8v1_A beadchips. Genotyping quality controls and imputation to the 1000 Genomes phase1_v3 March 2012 reference panel were carried out for the two chips separately and imputed data merged post-imputation. Prior to imputation variants were excluded because of missingness (call rate <98%), deviation from Hardy-Weinberg equilibrium ($P < 10^{-6}$) or low MAF

(<1%). Individuals were removed for poor overall call rates (<97%). In addition, excess heterozygosity, gender discrepancies and ethnic outliers in the study were removed. Genotypes were phased using SHAPEIT2 and imputation performed using the IMPUTE2 software.

Access to data from VIKING, ORCADES, CROATIA-KORCULA and CROATIA-SPLIT is via a managed open access procedure described at https://www.ed.ac.uk/mrc-human-genetics-unit/research/qtl-group. Researchers interested in working with these datasets should email: accessQTL@ed.ac.uk. The data and sample sharing process is facilitated by a full-time project manager and agreed proposals are conducted in collaboration with appropriate members of the QTL team.

2. Generating lead SNPs

The independent lead SNPs have been obtained from iterative clumping procedure in PLINK[18], which has been widely used[19]. First, we identify the SNP that has the minimum *p*-value from the single variant association analysis. This is the lead SNP for the first clump. Secondly, all SNPs with *p*-values less than 10^{-4}, in LD (defined as r^2 of 0.2) and within 500kb of the lead SNP, are clumped to the lead SNP of the first clump. Next, we repeat the previous two steps after excluding all SNPs from the first clump, this then becomes the second clump. This process is iteratively repeated until there are no more SNPs with a *p*-value less than 10^{-4}. The LD was calculated from the 1000 genomes phase 1 reference sample of European ancestry[20].

3. Gene expression and epigenetic resources

The Genotype-Tissue Expression (GTEx) project[21], provides publically accessible eQTL data for a range of cell and tissue types. Within our analysis we used specifically brain, bone and blood tissue/cell types for the analysis of Stratified LD score Regression[22], S-PrediXcan[23] and FUMA[1].

We furthermore extracted blood eQTL from the **Depression Genes and Networks (DGN)**[24] for S-PrediXcan[23] analyses.

The Roadmap Epigenomics consortium consists of 397 cell/tissue types from narrow peaks in six chromatin marks, DNase hypersensitivity and H3K27ac, H3K4me3, H3K4me1, H3K9ac, and H3K36me3 histone marks. For stratified LD score Regression[22] analysis, we restricted our analysis to 55 chromatin marks identified in neural and bone tissue/cell types.

Data from the ENCyclopedia Of DNA Elements (ENCODE) project[25] was used for gene expression and chromatin enrichment analysis, as part of a stratified LD score Regression[22]. We only used a subset of the epigenetic data on neural and bone tissues that match the neural and bone tissues from the GTEx project, but are from different donors. In total, there are 93 annotations from peaks for four chromatin marks; H3K27ac, H3K4me3, H3K4me1, and H3K36me3 for neural and bone tissues.

We used the **Biobank-Based Integrative Omics Study (BIOS) QTL browser**[26] for blood eQTL analysis via the web tool FUMA[1].

For eQTL analysis of brain tissue/cell types, we also used data from the **UK Brain Expression Consortium (UKBEC)**[27] via the web tool FUMA[1].

For chromatin interaction we used FUMA assigned chromatin interaction data, **A Compendium of Chromatin Contact Maps Reveal Spatially Active Regions in the Human Genome**[28].

4. Power analysis

Although this study has 80% power to detect a genetic effect explaining as little as 0.11% of the variance in HC (assuming a MAF of 0.02, an effect size of 0.22 for a standardized trait and a type I error of 3.3x10-8), it is less well powered to detect variation with lesser frequency in population-based samples. For example, the power to detect a genetic effect explaining as little as 0.05% (assuming a MAF of 0.005, an effect size of 0.22 for a standardized trait and a type I error of 3.3x10-8) is less than 3%[29].

5. Acknowledgments

Avon Longitudinal Study of Parents and Children (ALSPAC)

We are extremely grateful to all the families who took part in this study, the midwives for their help in recruiting them, and the whole ALSPAC team, which includes interviewers, computer and laboratory technicians, clerical workers, research scientists, volunteers, managers, receptionists and nurses. The UK Medical Research Council and Wellcome (Grant ref: 102215/2/13/2) and the University of Bristol provide core support for ALSPAC. This publication is the work of the authors who will serve as guarantors for the contents of this paper. A comprehensive list of grants funding is available on the ALSPAC website at (http://www.bristol.ac.uk/alspac/external/documents/grant-acknowledgements.pdf). Collection of HC data at age 15 years was supported by Wellcome and MRC (grant 076467/Z/05/Z to G.D.S). GWAS genotype data was generated by Sample Logistics and Genotyping Facilities at Wellcome Sanger Institute and LabCorp (Laboratory Corporation of America) using support from 23andMe. This study makes use of data generated by the cohorts arm of the UK10K consortium, which was supported by core Wellcome funding for UK10K (grant WT091310).

Generation R Study (GenR)

The Generation R Study is conducted by the Erasmus Medical Center in close collaboration with the School of Law and Faculty of Social Sciences of the Erasmus University Rotterdam, the Municipal Health Service Rotterdam area, Rotterdam, the Rotterdam Homecare Foundation, Rotterdam and the Stichting Trombosedienst & Artsenlaboratorium Rijnmond [STAR-MDC], Rotterdam. We gratefully acknowledge the contribution of children and parents, general practitioners, hospitals, midwives and pharmacies in Rotterdam. The generation and management of GWAS genotype data for the Generation R Study was done at the Genetic Laboratory of the Department of Internal Medicine, Erasmus MC, the Netherlands. We would like to thank Karol Estrada, Dr. Tobias A. Knoch, Anis Abuseiris, Luc V. de Zeeuw, and Rob de Graaf, for their help in creating GRIMP, BigGRID, MediGRID, and Services@MediGRID/D-Grid, [funded by the German Bundesministerium fuer Forschung und Technology; grants 01 AK 803 A-H, 01 IG 07015 G] for access to their grid computing resources. We thank Pascal Arp, Mila Jhamai, Marijn Verkerk, Lizbeth Herrera and Marjolein Peters for their help in creating, managing and QC of the GWAS database. We thank Jie Huang at the Wellcome Trust's Sanger Institute, at Hinxton, U.K. for the creation of imputed data, with the support of Marijn Verkerk, Carolina Medina-Gomez, PhD, and Anis Abuseiris and their input for the analysis setup. The general design of Generation R Study is made possible by financial support from the Erasmus Medical Center, Rotterdam, the Erasmus University Rotterdam, the Netherlands Organization for Health Research and Development (ZonMw), the Netherlands Organisation for Scientific Research (NWO), the Ministry of Health, Welfare and Sport and the Ministry of Youth and Families. Additionally, the Netherlands Organization for Health Research and Development supported authors of this manuscript (ZonMw 907.00303, ZonMw 916.10159, ZonMw VIDI 016.136.361 to V.W.J., and ZonMw VIDI 016.136.367 to F.R.). V.W.J. received a Consolidator Grant from the European Research Council (ERC-2014-CoG-648916). This project also received funding from the European Union's Horizon 2020 research and innovation programme under grant agreements No 633595 (DynaHEALTH) and No 733206 (LIFECYCLE). This publication is the work of the authors who will serve as guarantors for the contents of this paper.

Western Australian Pregnancy Cohort study (Raine)

This study was supported by the National Health and Medical Research Council of Australia [grant numbers 572613, 403981 and 003209] and the Canadian Institutes of Health Research [grant number MOP-82893]. The authors are grateful to the Raine Study participants and their families, and to the Raine Study research staff for cohort coordination and data collection. The authors gratefully acknowledge the NH&MRC for their long term contribution to funding the study over the last 29 years and also the following Institutions for providing funding for Core Management of the Raine Study: The University of Western Australia (UWA), Curtin University, Raine Medical Research Foundation, UWA Faculty of Medicine, Dentistry and Health Sciences, The Telethon Institute for Child Health ResearchKids Institute, and Women and Infants Research Foundation (King Edward Memorial Hospital), Murdoch University, The University of Notre Dame (Australia), and Edith

Cowan University. The authors gratefully acknowledge the assistance of the Western Australian DNA Bank (National Health and Medical Research Council of Australia National Enabling Facility). This work was supported by resources provided by the Pawsey Supercomputing Centre with funding from the Australian Government and the Government of Western Australia. This publication is the work of the authors who will serve as guarantors for the contents of this paper.

Copenhagen Prospective Study on Asthma in Children (COPSAC): COPSAC2000 and COPSAC2010
We express our deepest gratitude to the children and families of the COPSAC2000 and COPSAC2010 cohort studies for all their support and commitment. We acknowledge and appreciate the unique efforts of the COPSAC research team. All funding received by COPSAC is listed on www.copsac.com. The Lundbeck Foundation (Grant no R16-A1694); The Ministry of Health (Grant no 903516); Danish Council for Strategic Research (Grant no 0603-00280B) and The Capital Region Research Foundation have provided core support to the COPSAC research center. This publication is the work of the authors who will serve as guarantors for the contents of this paper.

Infancia y Medio Ambiente (INMA)
ISGlobal is a member of the CERCA Programme, Generalitat de Catalunya. This study was funded by grants from Instituto de Salud Carlos III (Red INMA G03/176; CB06/02/0041; PI041436; PI081151 incl. FEDER funds; PS09/00432; PI12/01890 incl. FEDER funds; CP13/00054 incl. FEDER funds, MS13/00054), CIBERESP, Generalitat de Catalunya-CIRIT 1999SGR 00241, Generalitat de Catalunya-AGAUR (2009 SGR 501, 2014 SGR 822), Fundació La marató de TV3 (090430), Spanish Ministry of Economy and Competitiveness (SAF2012-32991 incl. FEDER funds), Agence Nationale de Securite Sanitaire de l'Alimentation de l'Environnement et du Travail (1262C0010), EU Commission (261357, 308333 and 603794). This publication is the work of the authors who will serve as guarantors for the contents of this paper.

Hellenic Isolated Cohorts (HELIC)
This work was funded by the Wellcome Trust (098051) and the European Research Council (ERC-2011-StG 280559-SEPI). The MANOLIS cohort is named in honour of Manolis Giannakakis, 1978-2010. We thank the residents of the Mylopotamos villages, and of the Pomak villages, for taking part. The HELIC study has been supported by many individuals who have contributed to sample collection (including A. Athanasiadis, O. Balafouti, C. Batzaki, G. Daskalakis, E. Emmanouil, C. Giannakaki, M. Giannakopoulou, A. Kaparou, V. Kariakli, S. Koinaki, D. Kokori, M. Konidari, H. Koundouraki, D. Koutoukidis, V. Mamakou, E. Mamalaki, E. Mpamiaki, M. Tsoukana, D. Tzakou, K. Vosdogianni, N. Xenaki, E. Zengini), data entry (T. Antonos, D. Papagrigoriou, B. Spiliopoulou), sample logistics (S. Edkins, E. Gray), genotyping (R. Andrews, H. Blackburn, D. Simpkin, S. Whitehead), research administration (A. Kolb-Kokocinski, S. Smee, D. Walker) and informatics (M. Pollard, J. Randall). This publication is the work of the authors who will serve as guarantors for the contents of this paper.

Orkney Complex Disease Study (ORCADES)
The Orkney Complex Disease Study (ORCADES) was supported by the Chief Scientist Office of the Scottish Government (CZB/4/276, CZB/4/710), a Royal Society URF to J.F.W., the MRC Human Genetics Unit quinquennial programme "QTL in Health and Disease", Arthritis Research UK and the European Union framework program 6 EUROSPAN project (contract no. LSHG-CT-2006-018947). DNA extractions were performed at the Wellcome Trust Clinical Research Facility in Edinburgh. We would like to acknowledge the invaluable contributions of the research nurses in Orkney, the administrative team in Edinburgh, the people of Orkney, and the data analysts in particular Dr Thibaud Boutin for the genotype imputation to the HRC reference panel and Dr Peter Joshi for setting up the GWAS analysis pipeline used in this analysis. This publication is the work of the authors who will serve as guarantors for the contents of this paper.

10001 Dalmations: The Croatian Biobank (CROATIA)
The CROATIA studies were funded by grants from the Medical Research Council (UK), from the Republic of Croatia Ministry of Science, Education and Sports (108-1080315-0302; 216-1080315-0302) and the Croatian Science Foundation (8875); and the CROATIA-Korčula genotyping was funded by the European Union framework program 6 project EUROSPAN (LSHGCT2006018947).

We would like to acknowledge the invaluable contribution of Dr Ozren Polasek who led and managed the recruitment in both Korcula and Split, the recruitment team from the Croatian Centre for Global Health, University of Split for the data collection and all the participants. We are grateful to Peter Lichner and the Helmholtz Zentrum Munchen (Munich, Germany), AROS Applied Biotechnology, Aarhus, Denmark and the Edinburgh Clinical research facility (Edinburgh, United Kingdom) for SNP array genotyping. Genetic analyses were supported by the MRC HGU "QTL in Health and Disease" core programme. We acknowledge the contribution of Dr Thibaud Boutin for the HRC imputation of the CROATIA-Korcula cohort and establishment of the GWAS analysis pipeline, as well as Holly Trochet for performing genotype quality controls and imputation to the 1000G reference panel for the CROATIA-Split dataset. This publication is the work of the authors who will serve as guarantors for the contents of this paper.

The Viking Health Study – Shetland (VIKING)
The Viking Health Study – Shetland (VIKING) was supported by the MRC Human Genetics Unit quinquennial programme grant "QTL in Health and Disease". DNA extractions and genotyping were performed at the Edinburgh Clinical Research Facility, University of Edinburgh. We would like to acknowledge the invaluable contributions of the research nurses in Shetland, the administrative team in Edinburgh, the people of Shetland, and the data analysts in particular Dr Thibaud Boutin for the genotype imputation to the HRC reference panel and Dr Peter Joshi for setting up the GWAS analysis pipeline used in this analysis. This publication is the work of the authors who will serve as guarantors for the contents of this paper.

UConn Health research collaboration
We like to thank Dr. J. L. Cotney (Genetics & Genome Sciences, UConn Health) for a discussion of the functionalities of our HC GWAS hits with respect to craniofacial eQTL and mQTL.

Research Centre Jülich research collaboration
We would like to thank Dr. T.W. Mühleisen and Prof. S. Cichon (Department of Biomedicine, University Hospital Basel) for a discussion of the functionalities of our HC GWAS hits.

The Cohorts for Heart and Aging Research in Genomic Epidemiology (CHARGE) Consortium
We would like to thank the CHARGE consortium for making their summary statistics publicly available, and offering us the possibility to incorporate this information into our analysis.

The Enhancing NeuroImaging Genetics through Meta-Analysis (ENIGMA) Consortium
We would like to thank the ENIGMA Consortium for making their summary statistics publicly available, and offering us the possibility to incorporate this information into our analysis.

UKBiobank
This research has been conducted using the UK Biobank Resource under Application Number 16066. We thank all participants of the UK Biobank for their contribution.

Author funding
S.H and B.S.P are with a unit that receives funding from the University of Bristol and the UK Medical Research Council (grant ref MC_UU_12013/3). S.H receives support from Wellcome (grant ref 201237/Z/16/Z). N.J.T is a Wellcome Trust Investigator (202802/Z/16/Z), a work-package lead in the Integrative Cancer Epidemiology Programme (ICEP) that is supported by a Cancer Research UK programme grant (C18281/A19169) and works within the University of Bristol NIHR Biomedical Research Centre (BRC). T.J.C was funded by Medical Research Council grant MR/M012069/1. C.Y.S, S.E.F and B.S.P receives Core Support from the Max Planck Society.

LD Hub
We gratefully acknowledge all the studies and databases that made GWAS summary data available to LD Hub: ADIPOGen (Adiponectin genetics consortium), C4D (Coronary Artery Disease Genetics Consortium), CARDIoGRAM (Coronary ARtery DIsease Genome wide Replication and Meta-analysis), CKDGen (Chronic Kidney Disease Genetics consortium), dbGAP (database of Genotypes and Phenotypes), DIAGRAM (DIAbetes Genetics Replication And Meta-analysis), ENIGMA

(Enhancing Neuro Imaging Genetics through Meta Analysis), EAGLE (EArly Genetics & Lifecourse Epidemiology Eczema Consortium, excluding 23andMe), EGG (Early Growth Genetics Consortium), GABRIEL (A Multidisciplinary Study to Identify the Genetic and Environmental Causes of Asthma in the European Community), GCAN (Genetic Consortium for Anorexia Nervosa), GEFOS (GEnetic Factors for OSteoporosis Consortium), GIANT (Genetic Investigation of ANthropometric Traits), GIS (Genetics of Iron Status consortium), GLGC (Global Lipids Genetics Consortium), GPC (Genetics of Personality Consortium), GUGC (Global Urate and Gout consortium), HaemGen (haemotological and platelet traits genetics consortium), HRgene (Heart Rate consortium), IIBDGC (International Inflammatory Bowel Disease Genetics Consortium), ILCCO (International Lung Cancer Consortium), IMSGC (International Multiple Sclerosis Genetic Consortium), MAGIC (Meta-Analyses of Glucose and Insulin-related traits Consortium), MESA (Multi-Ethnic Study of Atherosclerosis), PGC (Psychiatric Genomics Consortium), Project MinE consortium, ReproGen (Reproductive Genetics Consortium), SSGAC (Social Science Genetics Association Consortium) and TAG (Tobacco and Genetics Consortium), TRICL (Transdisciplinary Research in Cancer of the Lung consortium), UK Biobank. We gratefully acknowledge the contributions of Alkes Price (the systemic lupus erythematosus GWAS and primary biliary cirrhosis GWAS) and Johannes Kettunen (lipids metabolites GWAS).

6. **Web resources**

Brain xQTL: http://mostafavilab.stat.ubc.ca/xQTLServe/

FUMA: http://fuma.ctglab.nl/

GSEM: https://gitlab.gwdg.de/beate.stpourcain/gsem

GWAVA: https://www.sanger.ac.uk/sanger/StatGen_Gwava

LDHub platform: http://ldsc.broadinstitute.org/

PAGEANT: https://andrewhaoyu.shinyapps.io/PAGEANT/

UCSC Genome Browser: https://genome.ucsc.edu/

UK-WHO growth charts: https://www.rcpch.ac.uk/child-health/research-projects/uk-who-growth-charts/uk-who-growth-chart-resources-0-4-years/uk-who-0

References

1. Watanabe, K., Taskesen, E., Bochoven, A. & Posthuma, D. Functional mapping and annotation of genetic associations with FUMA. *Nat Commun* **8**, 1826 (2017).
2. Adams, H.H.H. *et al.* Novel genetic loci underlying human intracranial volume identified through genome-wide association. *Nat Neurosci* **19**, 1569-1582 (2016).
3. Ikram, M.A. *et al.* Common variants at 6q22 and 17q21 are associated with intracranial volume. *Nat Genet* **44**, 539-+ (2012).
4. Elliott, L. *et al.* Genome-wide association studies of brain structure and function in the UK Biobank.Preprint at https://doi.org/10.1101/178806 (2018).
5. Stein, J.L. *et al.* Identification of common variants associated with human hippocampal and intracranial volumes. *Nat Genet* **44**, 552 (2012).
6. Hibar, D.P. *et al.* Common genetic variants influence human subcortical brain structures. *Nature* **520**, 224 (2015).
7. Taal, H.R. *et al.* Common variants at 12q15 and 12q24 are associated with infant head circumference. *Nat Genet* **44**, 532-+ (2012).
8. Boyd, A. *et al.* Cohort profile: the 'children of the 90s'—the index offspring of the Avon Longitudinal Study of Parents and Children. *Int J Epidemiol* **42**, 111-127 (2013).
9. Fraser, A. *et al.* Cohort profile: the Avon Longitudinal Study of Parents and Children: ALSPAC mothers cohort. *Int J Epidemiol* **42**, 97-110 (2012).
10. UK 10K Consortium. The UK10K project identifies rare variants in health and disease. *Nature* **526**, 82-90 (2015).
11. Huang, J. *et al.* Improved imputation of low-frequency and rare variants using the UK10K haplotype reference panel. *Nat Commun* **6**(2015).
12. Straker, L.M. *et al.* Rationale, design and methods for the 22 year follow-up of the Western Australian Pregnancy Cohort (Raine) Study. *BMC Public Health* **15**, 663 (2015).
13. Guxens, M. *et al.* Cohort profile: the INMA—INfancia y Medio Ambiente—(environment and childhood) project. *Int J Epidemiol* **41**, 930-940 (2011).
14. Bisgaard, H. The Copenhagen Prospective Study on Asthma in Childhood (COPSAC): design, rationale, and baseline data from a longitudinal birth cohort study. *Ann Allergy Asthma Immunol* **93**, 381-389 (2004).
15. Bisgaard, H. *et al.* Deep phenotyping of the unselected COPSAC2010 birth cohort study. *Clin Exp Allergy* **43**, 1384-1394 (2013).
16. McQuillan, R. *et al.* Runs of homozygosity in European populations. *Am J Hum Genet* **83**, 359-372 (2008).
17. Polašek, O. Future of biobanks–bigger, longer, and more dimensional. *Croatian med Jl* **54**, 496 (2013).
18. Purcell, S. *et al.* PLINK: a tool set for whole-genome association and population-based linkage analyses. *Am J Hum Genet* **81**, 559-575 (2007).
19. Okbay, A. *et al.* Genetic variants associated with subjective well-being, depressive symptoms, and neuroticism identified through genome-wide analyses. *Nat genet* **48**, 624-633 (2016).
20. 1000 Genomes Project Consortium. A global reference for human genetic variation. *Nature* **526**, 68 (2015).
21. GTEx Consortium. Genetic effects on gene expression across human tissues. *Nature* **550**, 204 (2017).
22. Finucane, H.K. *et al.* Heritability enrichment of specifically expressed genes identifies disease-relevant tissues and cell types. *Nat Genet* **50**, 621-629 (2018).
23. Barbeira, A.N. *et al.* Exploring the phenotypic consequences of tissue specific gene expression variation inferred from GWAS summary statistics. *Nat Commun* **9**, 1825 (2018).
24. Battle, A. *et al.* Characterizing the genetic basis of transcriptome diversity through RNA-sequencing of 922 individuals. *Genome res* **24**, 14-24 (2014).
25. Consortium, E.P. An integrated encyclopedia of DNA elements in the human genome. *Nature* **489**, 57-74 (2012).
26. Zhernakova, D.V. *et al.* Identification of context-dependent expression quantitative trait loci in whole blood. *Nat genet* **49**, 139 (2017).

27. Ramasamy, A. *et al.* Genetic variability in the regulation of gene expression in ten regions of the human brain. *Nat neurosci* **17**, 1418 (2014).
28. Schmitt, A.D. *et al.* A Compendium of Chromatin Contact Maps Reveal Spatially Active Regions in the Human Genome. *Cell rep* **17**, 2042-2059 (2016).
29. Derkach, A., Zhang, H. & Chatterjee, N. Power Analysis for Genetic Association Test (PAGEANT) provides insights to challenges for rare variant association studies. *Bioinformatics*, btx770 (2017).
