# Supplementary material for: Low-frequency variation in TP53 has large effects on head circumference and intracranial volume
Source: Nat Commun. 2019 Jan 21;10:357. doi: 10.1038/s41467-018-07863-x (PMC6341110; doi:10.1038/s41467-018-07863-x)
Supplement: Supplementary file 3 — Description of Additional Supplementary Files [file 41467_2018_7863_MOESM3_ESM.pdf]

Description of Additional Supplementary Files

Supplementary Data 1:	HC (Pediatric) meta-analysis: Independent single variants (p < 1.0E-04)
Supplementary Data 2:	HC (Adult) meta-analysis: Independent single variants (p < 1.0E-04)
Supplementary Data 3:	HC (Pediatric+adult) meta-analysis: Independent single variants (p < 1.0E-04)
Supplementary Data 4:	Genome-wide summary statistics for HC (Pediatric and adult), standardised beta-coefficients
Supplementary Data 5:	HC (Pediatric) meta-analysis: Gene-based analysis (multiple testing threshold p < 1.7E-06)
Supplementary Data 6:	HC (Adult) meta-analysis: Gene-based analysis (multiple testing threshold p< 1.7E-06)
Supplementary Data 7:	HC (Pediatric+adult) meta-analysis: Gene-based analysis (multiple testing threshold p < 1.7E-06)
Supplementary Data 8:	S-prediXcan for HC (Pediatric+adult) for cerebellum, cortex and whole blood
Supplementary Data 9:	LDHUB unconstrained LDSC genetic correlations for HC (Pediatric+adult)
Supplementary Data 10:	Stratified LDSC regression for HC (Pediatric+adult)
Supplementary Data 11:	ICV+HC (Pediatric+adult) meta-analysis: Novel independent single variants (p < 5E-08)
Supplementary Data 12:	ICV+HC (Pediatric+adult) meta-analysis: Known single variant signals for head circumference (HC), intracranial volume (ICV) and brain volume
Supplementary Data 13:	Genome-wide summary statistics for ICV+HC(Pediatric+adult), standardised beta-coefficients
Supplementary Data 14:	Genome-wide FUMA for HC (Pediatric+adult)
Supplementary Data 15:	Genome-wide FUMA for ICV+HC (Pediatric+adult)
Supplementary Data 16:	FUMA. SNP annotation: ICV+HC (Pediatric+adult): Novel independent single variants (p < 5E-08)
Supplementary Data 17:	FUMA. Genes mapped by SNPs: ICV+HC (Pediatric+adult): Novel independent single variants (p < 5E-08)
Supplementary Data 18:	FUMA GWAS catalogue: ICV+HC (Pediatric+adult): Novel independent single variants (p < 5E-08)
Supplementary Data 19:	Brain xQTL query for rs35850753. Information about rs78378222 was not available.
Supplementary Data 20:	UKBiobank PheWAS for rs78378222 and rs35850753 (multiple testing threshold p < 2.26E-06)
