# Supplementary material for: Low-frequency variation in TP53 has large effects on head circumference and intracranial volume
Source: Nat Commun. 2019 Jan 21;10:357. doi: 10.1038/s41467-018-07863-x (PMC6341110; doi:10.1038/s41467-018-07863-x)
Supplement: Supplementary file 2 — Peer Review Files [file 41467_2018_7863_MOESM2_ESM.pdf]

Reviewer #1 (Remarks to the Author):

This is an interesting and important study that performed genetic association analyses of the head circumference (HC), supplemented by meta-analysis of the intracranial volume (ICV) from two large imaging samples. There is an intimate and evolutionary strong relationship between HC and ICV. The authors should mention that humans are one of very few species that are subject to cerebro pelvic disproportion (CPD). This refers to the evolutionary push for upright locomotion that limited the size of the birthing canal for humans. More so than for other primates. This is a very strong evolutionary push because CPD was lethal to both mother and offspring until the cesarean section delivery became common. In other words, the majority of the human evolution was spent under this sword of ultimate death due to CPD. Therefore, humans, among all the primate species, are born with little myeline in their brain despite the clear evolutionary preference for having more able offspring at the point of birth. The authors should incorporate this into the discussion.

Another important aspect is that the HC at birth is only modestly related to the HC in adulthood. As nearly all the ICV change during development is driven by cerebral myelination, there has to be a genetic mechanism that regulate and synchronize these two processes. I know that this may be slightly outside of the scope of the paper, it should be discussed in the manuscript as a driving force of the ICV/HC change. This is especially important because P53 mutations are linked to the brain tumors of glial origin.

Reviewer #2 (Remarks to the Author):

The authors present novel GWAS data on head circumference (HC) in both pediatric and adult cohorts, identifying (and replicating) a novel locus at the TP53 gene. Results are then meta-analyzed with prior GWAS results for intracranial volume (ICV), a highly correlated phenotype, yielding several additional loci.

This study is clearly presented and reports important new data. However, a few critical questions should be addressed to clarify the interpretation of results:

1) It is not clear if the samples utilized in the HC study are fully independent from the cohorts utilized in the ICV study. If there is any sample overlap, then results of the meta-analysis would be inflated, and MTAG would be a more appropriate approach.

2) Assuming the results of the meta-analysis are valid, the reader is still left with ambiguity concerning which specific SNP at the TP53 locus is implicated. Greater details should be presented (including forest plots, conditional analysis, etc).

3) The additional loci in Table 6 are only briefly discussed. Each should be fully annotated in terms of genes and other relevant information (eQTL, other GWAS overlap, etc).

4) More broadly, assuming the results in Table 6 are accurate (ie, no cohort overlap), a full FUMA annotation would be helpful. Moreover, a comprehensive presentation of Predixcan results would be more appropriate than the selective presentation currently available.

5) The authors did not meta-analyze their results with those from the EGG Consortium. Why not? Are these overlapping?

Re: Manuscript NCOMMS_18_17208_T entitled "Low-frequency variation in *TP53* has large effects on head circumference and intracranial volume"

Thank you for considering this manuscript for publication in Nature Communication and for this constructive feedback. Please find a detailed response to each question raised by the reviewers underneath each comment.

Reviewer #1

1.1 This is an interesting and important study that performed genetic association analyses of the head circumference (HC), supplemented by meta-analysis of the intracranial volume (ICV) from two large imaging samples. There is an intimate and evolutionary strong relationship between HC and ICV. The authors should mention that humans are one of very few species that are subject to cerebro pelvic disproportion (CPD). This refers to the evolutionary push for upright locomotion that limited the size of the birthing canal for humans. More so than for other primates. This is a very strong evolutionary push because CPD was lethal to both mother and offspring until the cesarean section delivery became common. In other words, the majority of the human evolution was spent under this sword of ultimate death due to CPD. Therefore, humans, among all the primate species, are born with little myeline in their brain despite the clear evolutionary preference for having more able offspring at the point of birth. The authors should incorporate this into the discussion.

We thank the reviewer for this important suggestion and have adjusted the manuscript accordingly. We assume the reviewer refers to cephalopelvic (also known as fetopelvic) disproportion (CPD), i.e. the mismatch between the maternal pelvis (birth canal), and the fetal head[1]. The manuscript has been revised as follows:

Discussion, page 17

"*Genetic correlation analyses provided strong evidence for shared genetic determinants between HC and both anthropometric (birth weight, height, waist and hip circumference) and cognitive traits, as well as ICV. Genetic correlations with waist circumference and hip circumference recapitulate observed correlations between the size of the maternal pelvis and the size of the neonatal cranium*[2]*. This is important as a mismatch between the maternal pelvis and the fetal head*[1]*, i.e. a cephalopelvic disproportion (CPD, also known as fetopelvic disproportion), can put the lives of both mother and fetus at risk, if left untreated. Mathematical models show that evolutionary forces such as a weak directional selection for a large neonate and/or a weak selection for a narrow pelvis can account for the considerable incidence of CPD in humans*[3]*, and predict a further rise in CPD incidence due to the frequent use of Caesarian sections during recent years*."

We were interested in the hypothesis that the unusual myelin growth characteristics of humans is an adaptive response to CPD. We agree with the reviewer that myelinated axons (white matter tracts) account for a large proportion of central nervous system volume in mature humans (~40%), which is higher than observed for other species and likely to reflect the drive for an expanding neuronal connectivity during human brain evolution[4]. We furthermore acknowledge the evidence that myelin is histologically not present at birth (except in the cerebellum and brainstem)[5]. Myelinated axons grow predominantly postnatally with a lag period followed by exponential growth over the first 12–16 months and slower growth from 2 to 5 years of age[5]. However, we were unable to find evidence in the literature supporting a (causal) link between HC at birth, risk of CPD and myelin content of the brain, possibly because this hypothesis is novel and currently untested. We have therefore included a brief discussion of cortical myelin (detailed in the response to point 1.3), but have not extended this to discussing a link between CPD and myelin content in this manuscript.

1.2 Another important aspect is that the HC at birth is only modestly related to the HC in adulthood.

Thank you for this comment. While we provide evidence for age-specific genetic contributions to HC during childhood and adolescence using longitudinal modelling, these data do not extend into adulthood. We therefore estimated the genetic correlation between HC in infancy and HC (Adult) using LD-score regression method and Meta GWAS summary statistics[6] as r_g of 0.59 (SE=0.31). Thus, the true genetic correlation might be as low as -0.0176 (lower 95% confidence interval) or as high as 1.00 (upper 95% confidence interval, truncated at 1), assuming symmetrically distributed SE. Thus, we are unable to make any statements about the strength of the genetic correlation between HC at birth and HC in adulthood that are of high confidence. We have now revised the legend of Figure 5d to clearly indicate that bars represent standard errors around genetic correlation estimates:

"*...Linkage-disequilibrium score correlation (LDSC-r_g) for HC(Pediatric), HC(Adult) and HC(Pediatric+adult) and 235 phenotypes: 17 genetic correlation estimates passing a Bonferroni threshold (p<0.00014) are shown with their standard errors.*"

1.3 As nearly all the ICV change during development is driven by cerebral myelination, there has to be a genetic mechanism that regulate and synchronize these two processes. I know that this may be slightly outside of the scope of the paper, it should be discussed in the manuscript as a driving force of the ICV/HC change. This is especially important because P53 mutations are linked to the brain tumors of glial origin.

We thank the reviewer for this interesting discussion point. We noted that by 60 months of age, children's cerebral white matter contents, as assessed through myelin water fraction, approached approximately 80% of their adult values[5]. Thus, the majority of biological mechanisms (and their genetic counterparts) regulating and coordinating HC and brain growth through myelination will act outside the developmental window studied within our GWAS, and are thus beyond the scope of a targeted gene-based investigation. We are aware, however, that white matter and overall brain growth may continue into the second and third decade of life[5]. We speculated whether meninges may play a key role in the coordinated integration of signaling pathways regulating both neural and skeletal cranial growth, and have included these thoughts with respect to myelinated white matter within the Discussion of our manuscript:

Discussion, page 15/16

"*In particular, there is support from animal and tissue models for a role of p53 in neural crest cell (NCC) development*[7] *with NCCs supplementing head mesenchyme during fetal development* [8,9]. *NCCs also contribute to the development of a thick 3-membrane layer called the meninges, that cover the telencephalon*[8,9] *and directly locate underneath the skull. In particular, Pia mater, the innermost layer of the meninges, adheres closely to sulci and fissures of the cortex. During postnatal brain growth, including extensive increases in myelinated white matter*[5]*, the calvarial bones are drawn outward, partially due to the expanding meninges, triggering the production of membranous skull bone*[9]. *Consequently, meninges have been thought to play a key role in the coordinated integration of signaling pathways regulating both neural and skeletal cranial growth*[8]. *It is possible to speculate that p53 is part of these joint regulatory mechanisms, for example, via Wnt signaling regulation*[10]."

However, we are aware that these thoughts are highly speculative and have therefore described tumors associated with both rs78378222 and rs35850753 in more detail, in order to illustrate the breadth of potentially affected tissues and cell types:

Discussion, page 15

It is noteworthy that rs78378222 has been previously associated to risk of cancers including tumors of the nervous system such as glioma [11]*, a malignant tumor of glial tissue, possibly including neural stem cells, glial progenitors and astrocytes as cells of origin*[12]*, but also prostate cancer and colorectal adenoma*[13].

Both, rs35850753 and rs78378222 have also been robustly associated with neuroblastoma [14], a sympaticoadrenal lineage neural crest-derived tumor.

Reviewer #2

The authors present novel GWAS data on head circumference (HC) in both pediatric and adult cohorts, identifying (and replicating) a novel locus at the TP53 gene. Results are then meta-analyzed with prior GWAS results for intracranial volume (ICV), a highly correlated phenotype, yielding several additional loci. This study is clearly presented and reports important new data. However, a few critical questions should be addressed to clarify the interpretation of results:

2.1 It is not clear if the samples utilized in the HC study are fully independent from the cohorts utilized in the ICV study. If there is any sample overlap, then results of the meta-analysis would be inflated, and MTAG would be a more appropriate approach.

We would like to reassure the reviewer that there is no overlap between samples utilized in the HC Meta GWAS and the ICV samples recruited through the ENIGMA/CHARGE consortia[15]. For a detailed breakdown and comparison of contributing samples to HC and ICV analyses in our study, please see Table ICV_HC_Participating_cohorts.xlsx, incorporating information from Supplementary Table 1 by Adams *et al*[15]. This table has been uploaded for review only.

2.2 Assuming the results of the meta-analysis are valid, the reader is still left with ambiguity concerning which specific SNP at the TP53 locus is implicated. Greater details should be presented (including forest plots, conditional analysis, etc).

As suggested by the reviewer, we have now presented the genetic association between rs35850753 and HC (Pediatric + adult) and the genetic association between rs78378222 and ICV + HC (Pediatric+adult) meta-analyses (MA) in greater detail.

The forest plot for rs35850753 and other main signals of the HC (Pediatric + adult) MA are shown in Supplementary Figure S6. These plots show the effect size of each contributing HC sample, grouped by pediatric and adult samples, as well as the inverse-variance weighted MA estimate. Beta represents here the effect size in age-adjusted standard deviation units of HC, normalized across males and females.

We carefully thought about the request of the reviewer to provide forest plots for association signals of the ICV + HC (Pediatric+adult) MA. However, this analysis was performed combining summary statistics for ICV and HC using a weighted Z-score approach, as also the ICV GWAS MA carried out by Adams et al (2016) was conducted using a weighted Z-score approach. Beta coefficients for ICV + HC (Pediatric+adult) represent thus derived estimates, i.e. standardized regression coefficients based on Z-scores, effect allele frequencies and the standard deviation of the phenotype, as described in the Methods section of our manuscript. As forest plots are used to illustrate genetic effects from a fixed-effect inverse-variance weighted MA, we felt that these plots would be misleading, as the evidence for association between rs78378222 and ICV + HC (Pediatric+adult) was not assessed using this method. In addition, ICV and HC were assessed using scales with different physical properties (mm^3 versus cm, i.e. volumetric versus diametric), while forest plots depicting beta coefficients, as part of a fixed-effect inverse-variance weighted MA, suggest directly comparable effects. For these reasons, we have omitted forest plots depicting the association between rs78378222 and ICV + HC (Pediatric+adult) from the manuscript.

However, as suggested by the reviewer, we have performed conditional analyses of signals at rs35850753 and rs78378222, adjusted for each other, using GCTA software and HC (Pediatric+adult) and combined ICV + HC (Pediatric+adult) summary statistics respectively.

When conditioning the genetic signal at rs35850753 (lead SNP for HC) for variation at rs78378222, the statistical evidence for association with the rare T allele was strongly attenuated, but not fully abolished at

the nominal level (rs35850753: unconditional β = 0.22 (SE=0.038), p=2x10^{-8}; conditional β = 0.06 (SE=0.025), p=0.013). Reciprocally, the genetic signal at rs78378222 (lead SNP for ICV+HC) conditional on rs35850753 remained detectable at the nominal level (rs78378222: unconditional β = 0.18 (SE=0.027), p=7.9x10^{-11}; conditional β = 0.051 (SE=0.017), p=0.0024), based on analyses using standardized regression estimates.

We have adjusted the manuscript as follows:

Results: page 11

"To study the independence of the two signals, we carried out conditional analyses. Adjusting rs35850753 for variation at rs78378222, the association with HC (Pediatric+adult) was strongly attenuated, but remains present at the nominal level (conditional β = 0.06 (SE=0.025), p=0.013). Reciprocally, the signal at rs78378222, conditional on rs35850753, was still detectable at the nominal level in the combined cranial dimension analysis (conditional β = 0.051 (SE=0.017), p=0.0024), based on standardized regression estimates"

Methods: page 24

"To disentangle lead signals observed in both, the HC (Pediatric+adult) and the combined ICV+HC (Pediatric+adult) meta-analysis, variants were conditioned on each other using GCTA software and summary statistics."

The attenuation of association signals at both SNPs, once adjusted for each other, is unsurprising, given that both markers are in partial linkage disequilibrium (LD r^2=0.56). However, the existence of residual (albeit weakened) evidence for association in conditional analysis, taken in conjunction with the different functional characteristics of these markers, suggests that these may be tagging similar but non-identical functional biology at the *TP53* locus. These thoughts are included in the Discussion of our manuscript.

Discussion: page 15

"Thus, rare effect alleles at both rs78378222 and rs35850753 could potentially, via different biological mechanisms, be linked to impaired p53 activity and thus heightened proliferative potential and less apoptosis of normal human cells, consistent with larger HC scores and a larger cranial dimension."

2.3 The additional loci in Table 6 are only briefly discussed. Each should be fully annotated in terms of genes and other relevant information (eQTL, other GWAS overlap, etc).

Thank you for this suggestion, which we believe relates to the main table of novel single variant associations (Table 2). This table has now been expanded with a column indicating the gene nearest the lead signal. A complete list of mapped genes (based on variants in LD with the lead signal) has been previously provided in Supplementary Table S15. Please also see the response to comment 2.4, below.

In the combined HC + ICV analysis, we aimed to identify loci that are relevant to both underlying traits, reflecting an underlying cranial dimension. To identify these variants, we focused on single-variant signals that show stronger evidence for association in the combined analysis than in the single trait analysis. This identified several novel signals passing the GWAS threshold and highlighted several previously identified GWAS signals for either HC or ICV, with increased evidence for association. Novel signals have now been annotated in more detail, including eQTL and overlap with other GWAS findings, using FUMA (see below).

As noted above, we opted for a Z-weighted meta-analysis to identify novel variants of an underlying cranial dimension, as also the ICV GWAS MA carried out by Adams *et al* (2016)[15] was conducted using a weighted Z-score approach (providing no beta estimates). As mentioned above, beta coefficients for the

ICV + HC (Pediatric+adult) meta-analysis in this study represent derived estimates, i.e. standardized regression coefficients based on Z-scores, effect allele frequencies and the standard deviation of the phenotype, as described in the Methods section of our manuscript. Thus, also FUMA analyses of HC+ICV were carried out using standardized regression coefficients, and findings have to be interpreted with care.

Conceptually, we expect to identify variants that are shared between ICV and HC, together with variants that show trait-specific effects. However, trait-specific effects (in combination with a null effect from the other trait) are difficult to distinguish from null effects affecting both traits, due to the lack of heterogeneity statistics for Z-scores. Even when using derived beta estimates, heterogeneous effects would have been difficult to identify, as heterogeneity statistics such as Cochran's Q and I^2 often have low power, especially when the number of studies is small[16,17], comparing here ICV against HC. Thus, we cannot interpret signals that do not pass our selection criteria with respect to a shared underlying cranial dimension. In addition, ICV summary statistics from ENIGMA/CHARGE consortia were supplied to us following a correction for genomic control, which prevents an accurate estimation of LDSC-h^2 or the partitioning of such SNP-h^2.

We therefore omitted analyses utilizing SNP-trait associations at a genome-wide level, as they may be difficult to interpret, including those by LDSC or S-PrediXcan.

Submitted summary statistics and analyses can be found in full here:

https://archive.mpi.nl/islandora/object/lat%3A1839_3ab10f18_07d4_4171_8136_d0e0d100289f

Including the GWAS summary statistics, our manuscripts provides now the following Supplementary Data, as detailed in Supplementary Note 6:

Supplementary Data 1 meta-GWAS summary statistics
(**Link to GWAS folder**, referred to in Supplementary Tables S4, S15, S16)

Meta-analysis for HC (Pediatric +adult):
HC_entire_SumStat_240518.txt.gz

Meta-analysis for ICV+HC (Pediatric +adult):
HC_ICV_SumStat_240518.txt.gz

Supplementary Data 2 S-prediXcan
(**Link to analysis subfolder**, referred to in Supplementary Table S11)

S-PrediXcan with tissue from cerebellum for HC (Pediatric +adult):
S-prediXcan_cerebellum_HC(Pediatric +adult)

S-PrediXcan with tissue from cortex for HC (Pediatric +adult):
S-prediXcan_cortex_HC(Pediatric +adult)

S-PrediXcan with tissue from whole blood for HC (Pediatric +adult):
S-prediXcan_whole_blood_HC(Pediatric +adult)

Supplementary Data 3 LDSC correlations
(Link to analysis subfolder, referred to in Supplementary Table S14)
LDSC genetic correlations for HC (Pediatric +adult):

LDHUB_rg_HC(Pediatric +adult)

Supplementary Data 4: Stratified LDSC regression
(Link to analysis subfolder, referred to in Manuscript)
Stratified LDSC regression for HC (Pediatric +adult):
Stratified_LDSC_regression_HC(Pediatric+adult)

Supplementary Data 5: FUMA
(Link to analysis subfolder, referred to in Supplementary Tables S17, S18, S19)
FUMA for HC (Pedatric +adult):
FUMA_HC(Pediatric +adult)

FUMA for ICV+HC (Pediatric +adult):
FUMA_ICV_HC(Pediatric +adult)

We have updated the manuscript as follows:

Results: page 12

"A detailed variant annotation of all novel signals for the combined ICV and HC meta-analysis, including overlapping signals from the HC meta-analysis alone, was carried out using the FUMA webtool[18] (Supplementary Data 5) including variant annotation (Supplementary Table S17), mapped genes (Supplementary Table S18) and previously published studies (Supplementary Table S19)."

In addition, we now present also CADD scores and eQTL for the strongest novel HC and ICV+HC signals:

Results page 12:

"Species comparison showed that variation at rs78378222 is highly conserved (GERP-score=5.28)[19] and also predicted to be deleterious (CADD=17.97)[20], while variation at rs35850753 is not (GERP-score=-2.8; CADD=1.04) (Figure 3b, Supplementary Table S17)."

Results page 13:

"Furthermore, we identified variants in LD with the two cranial dimension signals at 1q44 and at 2p25.1 respectively that are predicted to be deleterious. rs12408455 (r^2 with rs2168812 = 0.99) is an intronic SNP within AKT3 (CADD = 17.87) and rs112040334 (r^2 with rs4513262 = 0.91) an intronic 11-bp insertion/deletion within KLF11 (CADD = 17.22, Supplementary Table S17). Both variants are in LD (r^2 > 0.6) with eQTL in blood (Supplementary Table S18), with the effect allele at both variants being associated with increasing transcript levels of AKT3 and KLF11 respectively. The variant with the highest CADD score identified in the combined ICV and HC meta-analysis is rs41288837. This low-frequency missense variant at 2p11.2 within TCF7L1 is predicted to belong to the 0.5% most deleterious substitutions in the human genome (MAF = 0.03, CADD = 24.30, Table 2, Supplementary Table S17). We observed no evidence for associated eQTL in blood or brain for this variant (Supplementary Table S18).

Notably, many genes containing SNPs in LD (r^2 > 0.6) with lead variants from the combined ICV and HC meta-analysis show mapped chromatin interactions within mesenchymal and human embryonic stem cells and mesoendoderm (Supplementary Table S18), including TP53."

Discussion, page 16:

"This includes the low-frequency variant rs41288837, predicted to belong to the 0.5% most deleterious substitutions in the human genome."

Discussion, page 17:

"Both, rs2168812 and rs3217870, have also been associated with cancer risk, similarly to the two TP53 variants, with the AKT3 variant rs12076373 (LD-r^2 with rs2168812: 0.70) being related to risk for Non-glioblastoma[11] and the CCND2 variant rs3217901 (LD-r^2 with rs3217870: 0.63) being related to colorectal cancer (Supplementary Table S19). Notably, AKT3 signaling is an essential intracellular pathway controlling neural crest development[21], and tissue-specific chromatin interactions in mesenchymal stem cells are reported for several novel loci (Supplementary Table S18), supporting a role of neural crest related processes in shaping final cranial dimension. "

2.4) More broadly, assuming the results in Table 6 are accurate (ie, no cohort overlap), a full FUMA annotation would be helpful. Moreover, a comprehensive presentation of Predixcan results would be more appropriate than the selective presentation currently available.

Thank you for this suggestion. The relevant FUMA analyses for both, HC (Pediatric + adult) and ICV + HC (Pediatric+adult), including eQTL and GWAS overlap for all novel loci, have been deposited in full in a publically available archive, alongside other follow-up analyses including full PrediXcan results (HC (Pediatric + adult) only) (see point 2.3).

As discussed above (2.3), methods requiring information on genome-wide association were only applied to HC analyses, and not the ICV+HC combined meta-analysis.

2.5) The authors did not meta-analyze their results with those from the EGG Consortium. Why not? Are these overlapping?

There were several reasons, why the results for HC (Pediatric+adult) were not combined with findings from the previously published MA on infant HC, carried out by the EGG consortium[22]. First, there was a large degree of sample overlap, including the ALSPAC, GenR, Raine and COPSAC cohorts. However, a fixed-effect inverse-weighted meta-analysis cannot account for this sample overlap.

Second, alternative analysis techniques that would allow for sample overlap, such as MTAG[23] are based on assumptions that may prevent an accurate identification of association with less frequent variants. Specifically, the minimum minor allele frequency (MAF) of our study (0.0025) is lower than the current filter of MAF >0.01 used by MTAG. MTAG furthermore relies on LDSC derived estimates, assuming that Hapmap3 SNPs with a MAF of 5% and higher, are sufficient to capture LD patterns between all studied variants. In addition, observed effects sizes at less frequent variants, such as those identified at *TP53*, are considerably larger than those identified within typical GWAS, with effects as large as a fifth of a SD. Those variants are typically excluded within MTAG[23]. Thus, less frequent variants are likely to follow a different effect size distribution compared to common variants [MAF>0.01], both in terms of the variance and covariance of their effect sizes, that may not very well captured by MTAG, see also[23].

Third, the previously published infant HC MA is based on an older imputation template (Hapmap 3) with limited coverage and little information on less frequent variation. Finally, the focus of this project was in characterizing genetic contributions to HC in adults and older children.

A new infant HC MA is currently carried out by our collaborators at GenR and we are happy to perform a MTAG meta-analysis, including data from all available studies on HC, based on MAF > 0.01, once these new summary statistics become publicly available.

Additional note to the editor

We have stored genome-wide summary statistics and functional analyses of this study in the Language Archive. The language archive is public repository (https://archive.mpi.nl, contact: Paul Trilsbeek Paul.Trilsbeek@mpi.nl) that allows storing highly diverse types of summary data, including those that have been generated as part of this publication.

There are more than 300 depositors from institutions around the world, and many more users accessing the data. The archive is a certified centre of the CLARIN European Research Infrastructure for Language Resources and Technology (https://www.clarin.eu/node/3822) and is a member of the ICSU World Data System (http://www.icsu-wds.org/community/membership/regular-members). Furthermore, the Language Archive has undergone the Data Seal of Approval assessment (https://assessment.datasealofapproval.org/assessment_163/seal/html/) and is currently awaiting the results of the application for Core Trust Seal (https://www.coretrustseal.org).

References

1. Maharaj, D. Assessing Cephalopelvic Disproportion: Back to the Basics. **65**, 387-395 (2010).
2. Neubauer, S. & Hublin, J.-J. The evolution of human brain development. *Evolutionary Biology* **39**, 568-586 (2012).
3. Mitteroecker, P., Huttegger, S., Fischer, B. & Pavlicev, M. Cliff-edge model of obstetric selection in humans. *Proceedings of the National Academy of Sciences of the United States of America* (2016).
4. Stassart, R.M., Möbius, W., Nave, K.-A. & Edgar, J.M. The Axon-Myelin Unit in Development and Degenerative Disease. **12**(2018).
5. Deoni, S.C.L., Dean, D.C., O'Muircheartaigh, J., Dirks, H. & Jerskey, B.A. Investigating white matter development in infancy and early childhood using myelin water faction and relaxation time mapping. *Neuroimage* **63**, 1038-1053 (2012).
6. Bulik-Sullivan, B. *et al.* An atlas of genetic correlations across human diseases and traits. *Nature Genetics* **47**, 1236-+ (2015).
7. Rinon, A. *et al.* p53 coordinates cranial neural crest cell growth and epithelial-mesenchymal transition/delamination processes. *Development* **138**, 1827-1838 (2011).
8. Richtsmeier, J.T. & Flaherty, K. Hand in glove: brain and skull in development and dysmorphogenesis. *Acta neuropathologica* **125**, 469-489 (2013).
9. Jin, S.-W., Sim, K.-B. & Kim, S.-D. Development and growth of the normal cranial vault: an embryologic review. *Journal of Korean Neurosurgical Society* **59**, 192 (2016).
10. Kim, N.H. *et al.* p53 and microRNA-34 are suppressors of canonical Wnt signaling. *Sci. Signal.* **4**, ra71-ra71 (2011).
11. Melin, B.S. *et al.* Genome-wide association study of glioma subtypes identifies specific differences in genetic susceptibility to glioblastoma and non-glioblastoma tumors. *Nature genetics* **49**, 789 (2017).
12. Zong, H., Verhaak, R.G.W. & Canoll, P. The cellular origin for malignant glioma and prospects for clinical advancements. *Expert Review of Molecular Diagnostics* **12**, 383-394 (2012).
13. Stacey, S.N. *et al.* A germline variant in the TP53 polyadenylation signal confers cancer susceptibility. *Nature genetics* **43**, 1098-1103 (2011).
14. Diskin, S.J. *et al.* Rare variants in TP53 and susceptibility to neuroblastoma. *Journal of the National Cancer Institute* **106**, dju047 (2014).
15. Adams, H.H.H. *et al.* Novel genetic loci underlying human intracranial volume identified through genome-wide association. *Nat Neurosci* **19**, 1569-1582 (2016).
16. von Hippel, P.T. The heterogeneity statistic I(2) can be biased in small meta-analyses. *BMC Medical Research Methodology* **15**, 35 (2015).

17. Higgins, J.P.T., Thompson, S.G., Deeks, J.J. & Altman, D.G. Measuring inconsistency in meta-analyses. *BMJ : British Medical Journal* **327**, 557-560 (2003).
18. Watanabe, K., Taskesen, E., van Bochoven, A. & Posthuma, D. Functional mapping and annotation of genetic associations with FUMA. *Nature Communications* **8**, 1826 (2017).
19. Davydov, E.V. *et al.* Identifying a high fraction of the human genome to be under selective constraint using GERP++. *PLoS computational biology* **6**, e1001025 (2010).
20. Kircher, M. *et al.* A general framework for estimating the relative pathogenicity of human genetic variants. *Nature genetics* **46**, 310-315 (2014).
21. Sittewelle, M. & Monsoro-Burq, A.H. AKT signaling displays multifaceted functions in neural crest development. *Developmental Biology* (2018).
22. Taal, H.R. *et al.* Common variants at 12q15 and 12q24 are associated with infant head circumference. *Nature Genetics* **44**, 532-+ (2012).
23. Turley, P. *et al.* Multi-trait analysis of genome-wide association summary statistics using MTAG. *Nature Genetics* (2018).

Reviewer #1 (Remarks to the Author):

The authors have adequately addressed my comments.

Reviewer #2 (Remarks to the Author):

The authors have successfully addressed the critiques, resulting in a more clear and more informative manuscript
